# Health and Economic Outcomes of Offering Buprenorphine in Homeless Shelters in Massachusetts

**DOI:** 10.1001/jamanetworkopen.2024.37233

**Published:** 2024-10-16

**Authors:** Avik Chatterjee, Emily A. Stewart, Sabrina A. Assoumou, Stavroula A. Chrysanthopoulou, Hana Zwick, Rebecca Arden Harris, Ryan O’Dea, Bruce R. Schackman, Laura F. White, Benjamin P. Linas

**Affiliations:** 1 Grayken Center for Addiction, Clinical Addiction Research and Education Unit, Section of General Internal Medicine, Department of Medicine, Boston Medical Center and Boston University Chobanian & Avedisian School of Medicine, Boston, Massachusetts; 2 Section of Infectious Diseases, Department of Medicine, Boston Medical Center and Boston University Chobanian & Avedisian School of Medicine, Boston, Massachusetts; 3 Department of Biostatistics, Brown University School of Public Health, Providence, R; 4 Department of Family Medicine and Community Health, Perelman School of Medicine, University of Pennsylvania Penn Presbyterian Medical Center, Philadelphia; 5 Department of Population Health Sciences, Weill Cornell Medicine, New York, New York; 6 Department of Biostatistics, Boston University School of Public Health, Boston, Massachusetts

## Abstract

**Importance:**

Overdose is the leading cause of death among people experiencing homelessness (PEH), but engagement in medication treatment is low in this population. Shelter-based buprenorphine may be a strategy for increasing initiation and retention on lifesaving medications.

**Objective:**

To estimate clinical outcomes and conduct an economic analysis of statewide shelter-based opioid treatment in Massachusetts.

**Design, Setting, and Participants:**

This economic evaluation study in Massachusetts used a cohort state-transition simulation model. Two cohorts were modeled starting in 2013, including (1) a closed cohort of a fixed population of PEH with history of high-risk opioid use over their lifetimes and (2) an open cohort in which membership could change over time, allowing assessment of population-level trends over a 10-year period. Data analysis occurred from January 2023 to April 2024.

**Exposures:**

Model exposures included (1) no shelter-based buprenorphine (status quo) and (2) offering buprenorphine in shelters statewide.

**Main Outcomes and Measures:**

Outcomes included overdose deaths, quality-adjusted life-years (QALYs) gained, and health care and modified societal perspective costs. Sensitivity analyses were conducted on key parameters.

**Results:**

In the closed cohort analysis of 13 800 PEH (mean [SD] age, 40.4 [13.1] years; 8749 male [63.4%]), shelter-based buprenorphine was associated with an additional 65.4 person-weeks taking buprenorphine over an individual’s lifetime compared with status quo. Shelter-based buprenorphine was cost saving when compared with the status quo, with a discounted lifetime cost savings from the health sector perspective of $1300 per person, and 0.2 additional discounted QALYs per person and 0.9 additional life-years per person. In the population-level simulation, 254 overdose deaths were averted over the 10-year period with the shelter-based buprenorphine strategy compared with the status quo (a 9.2% reduction of overdose deaths among PEH in Massachusetts). Overdose-related and other health care utilization undiscounted costs decreased by $3.0 million and $66.4 million, respectively. Shelter-based opioid treatment generated $44.7 million in additional medication and clinical costs, but saved $69.4 million in overdose and other health costs.

**Conclusions and Relevance:**

In this economic evaluation of clinical and economic outcomes among PEH, shelter-based buprenorphine was associated with fewer overdose deaths and was cost saving. These findings suggest that broad rollout of shelter-based buprenorphine may be an important tool in addressing the overdose crisis.

## Key Points

**Question:**

What are the costs and cost-effectiveness of an intervention offering buprenorphine to people experiencing homelessness in shelter-based clinics across Massachusetts?

**Findings:**

This economic evaluation modeling the natural history of opioid use disorder in a simulated cohort of 13 800 people experiencing homelessness found that a shelter-based buprenorphine strategy was associated with 254 fewer overdose deaths (a 9.2% reduction) over a 10-year period and was less costly than the status quo. The strategy generated $44.7 million in additional medication and clinical costs, but saved $69.4 million in overdose and other health costs.

**Meaning:**

These findings suggest that shelter-based buprenorphine may be an important tool for addressing the overdose crisis.

## Teaser

This economic evaluation study uses a simulation model to assess clinical and economic outcomes of a statewide shelter-based opioid treatment program among people experiencing homelessness in Massachusetts.

## Introduction

The high prevalence of opioid use disorder (OUD) among people experiencing homelessness (PEH) contributes to an elevated risk of opioid overdose,^1^ with opioid-related overdoses being the leading cause of death among PEH.^2,3^ Medications for OUD (MOUD) reduce the risk of overdose death.^4^ However, MOUD uptake is low among PEH^5^ due to numerous barriers to health care access.^6,7,8^ However, when PEH initiate MOUD and are engaged with care in tailored office-based opioid treatment, MOUD retention can be high^9^ and risk of death can decrease.^10^

Innovative models of care can increase MOUD access among PEH. Mobile MOUD programs, for example, improve buprenorphine access by bringing prescribers to PEH.^11,12^ Similarly, a recent simulation modeling study^13^ demonstrated that colocating buprenorphine access with syringe service programs—community-based harm reduction programs whose clientele has a high homelessness prevalence—can be a cost-effective approach to averting overdose deaths.^14^

Shelter-based buprenorphine prescribing is another strategy for increasing MOUD initiation and reducing mortality in this vulnerable population. Early findings from shelter-based opioid treatment (SBOT) programs in the Boston area show promising outcomes.^6,15^ In an evaluation of office-based addiction treatment at Boston Health Care for the Homeless Program,^10^ initiating buprenorphine at a shelter or outreach site (compared with an outpatient clinic) was associated with an approximately 40% lower mortality hazard. The goal of the current study was to estimate the long-term, population-level outcomes and cost-effectiveness of expanding SBOT statewide to the more than 100 emergency shelters in Massachusetts.^16^

## Methods

### RESPOND Model Structure

**Figure 1.**
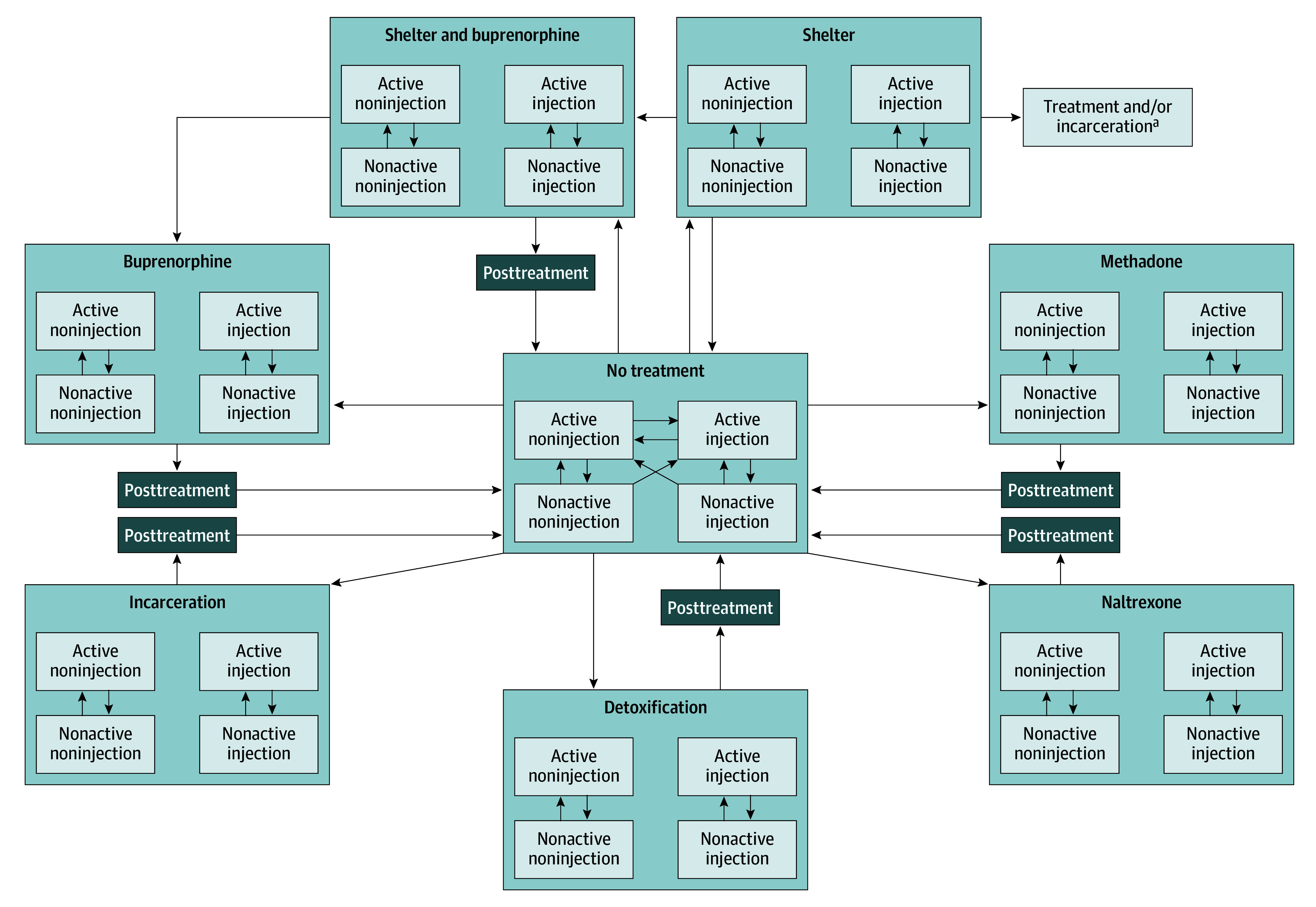
Researching Effective Strategies to Prevent Opioid Death Care Delivery (RESPOND) Module With Project-Specific Strategies Represented ^a^People in the shelter block can transition to all medications for opioid use disorder treatment, detoxification, and incarceration blocks.

This economic evaluation study was determined to be exempt from review and the requirement of informed consent by the Boston University Medical Campus and Boston Medical Center institutional review board. The reporting of the study followed the Consolidated Health Economic Evaluation Reporting Standards (CHEERS) reporting guideline. We utilized the Researching Effective Strategies to Prevent Opioid Death (RESPOND) model, a dynamic population, state-transition simulation model of OUD in Massachusetts, to simulate a population of PEH with OUD, projecting clinical outcomes and conducting an economic analysis estimating costs and cost-effectiveness of implementing SBOT programs in shelters across the state. The RESPOND model, described in detail in previous work^17^ and in eAppendix 1 and eAppendix 2 in Supplement 1, simulates the lifetime progression of a population living with OUD as a series of transitions between health states (Figure 1 and eAppendix 2 in Supplement 1).

The model captures the relapsing-remitting nature of OUD with 4 health states representing active vs nonactive and injection vs noninjection opioid use (eAppendix 3 in Supplement 1). Throughout the simulation, the population actively using opioids may enter one of the following treatment paths: no treatment, detoxification (medically supervised withdrawal), and outpatient MOUD (buprenorphine-naloxone, methadone, or extended-release injection naltrexone) (Table 1 and eAppendix 3 in Supplement 1). In every weekly time step, the population engaged with MOUD may continue treatment, overdose and remain in treatment, die from overdose or from another cause, or disengage from care (see eAppendix 3 and eAppendix 4 in Supplement 1 for details). During MOUD treatment, there is bidirectional movement between active and nonactive drug-use states, with a bias toward nonactive drug use and, subsequently, less health care utilization (eAppendix 3 in Supplement 1).^28^ Each MOUD has an independent association with overdose rate (eAppendix 3 in Supplement 1).^29,30^ Upon disengaging from MOUD, individuals move through a posttreatment state with elevated overdose risk. The population also faces an incarceration risk, which is higher for people actively using drugs (eAppendix 3 in Supplement 1).^31^ During incarceration, we assume the potential for continued drug use,^32^ and individuals face an elevated overdose risk upon release.^33^

**Table 1.** Model Parameters

Parameter	Base case value	Range evaluated^a^	Source
Population demographics and epidemiology at baseline, No./total No. (%)			
Sex			
Male	8749/13 800 (63.4)	NA	Fine et al, 2022^3^
Female	5051/13 800 (36.6)		
Age mean (SD), y	40.4 (13.1)	NA	Fine et al, 2022^3^
IDU	3450/13 800 (0.25)	NA	MA PHD^18^
Active drug use	12 558/13 800 (0.91)	NA	MA PHD^18^
SMR for IDU	8.09	3.24-8.09	MA PHD^18^ and Madushani et al, 2019^19^
SMR for non-IDU	3.27	1.31-3.27	PHD^18^ and Madushani et al, 2019^19^
Transition, monthly rate per 1000 people			
Shelter buprenorphine from shelter no treatment	50.18	4.99-50.18	Fine et al, 2021^10^
Shelter buprenorphine from no treatment	4.56	0.46-4.56	Fine et al, 2022^10^ and Hao et al,2022^20^
Community buprenorphine from shelter buprenorphine	981.05	87.96-981.05	Taylor et al, 2023^21^
Posttreatment from shelter buprenorphine	502.86	502.86-2558.72	Taylor et al, 2023^21^
To and from no treatment and shelter no treatment	363.64	NA	Hao et al,2022^20^
Overdose, monthly rate per 1000 people, No. (95% UI)^b^			
No treatment and shelter no treatment			
IDU	11.26 (11.21-11.31)	5.63-16.89	MA PHD^18^
Non-IDU	1.87 (1.86-1.88)	0.93-2.80	MA PHD^18^
Buprenorphine and shelter buprenorphine			
IDU	4.52 (4.49-4.55)	2.26-6.78	MA PHD^18^
Non-IDU	0.75 (0.75-0.75)	0.38-1.13	MA PHD^18^
Naltrexone			
IDU	7.82 (7.71-7.93)	3.91-11.73	MA PHD^18^
Non-IDU	1.30 (1.28-1.32)	0.65-1.95	MA PHD^18^
Methadone			
IDU	7.92 (7.82-8.01)	3.96-11.87	MA PHD^18^
Non-IDU	1.31 (1.30-1.33)	0.66-1.97	MA PHD^18^
Fatal overdoses, proportion of total overdoses (95% UI)^b^	0.3061 (0.3058-0.3065)	0.15-0.46	MA PHD^18^ and Roncarti et al, 2018^22^
Weekly pharmaceutical cost, $			
Buprenorphine	58.02	NA	McCollister et al, 2018^23^ and Murphy et al, 2019^24^
Methadone	5.13	NA	McCollister et al, 2018^23^ and Murphy et al, 2019^24^
Naltrexone	360.40	NA	McCollister et al, 2018^23^ and Murphy et al, 2019^24^
Shelter buprenorphine	58.02	NA	McCollister et al, 2018^23^ and Murphy et al, 2019^24^
Weekly treatment utilization cost, $			
Buprenorphine	77.71	NA	McCollister et al, 2018^23^ and Murphy et al, 2019^24^
Methadone	147.02	NA	McCollister et al, 2018^23^ and Murphy et al, 2019^24^
Naltrexone	29.02	NA	McCollister et al, 2018^23^ and Murphy et al, 2019^24^
Shelter buprenorphine	103.18	103.18-481.03	McCollister et al, 2018^23^; Murphy et al, 2019^24^; Hao et al, 2022^25^; and US BLS^26^
Weekly overdose cost, $			
Fatal overdose cost	1021.93	511.00-1532.98	McCollister et al, 2018^23^ and Murphy et al, 2019^24^
Nonfatal overdose cost	5428.26	2714.29-8142.85	McCollister et al, 2018^23^ and Murphy et al, 2019^24^
Weekly health care utilization cost, mean (range), $			
Healthcare sector perspective cost			
No treatment and shelter no treatment	373.09 (193.23-591.17)	186.55-559.63	McCollister et al, 2018^23^ and Murphy et al, 2019^24^
Buprenorphine and shelter buprenorphine	264.16 (125.07-429.99)	132.08-396.24	McCollister et al, 2018^23^ and Murphy et al, 2019^24^
Methadone	264.22 (125.23-429.90)	132.12-396.34	McCollister et al, 2018^23^ and Murphy et al, 2019^24^
Naltrexone	338.14 (170.33-547.91)	169.09-507.22	McCollister et al, 2018^23^ and Murphy et al, 2019^24^
Corrections	0	NA	McCollister et al, 2018^23^ and Murphy et al, 2019^24^
Modified societal perspective cost			
No treatment and treatment blocks, $	0	NA	NA
Corrections, $	1000.52	NA	US Federal Register^27^

Abbreviations: BLS, Bureau of Labor Statistics; IDU, injection drug use; MA PHD, Massachusetts Public Health Data Warehouse; NA, not applicable; SMR, standardized mortality ratio; UI, uncertainty interval.

^a^ A probabilistic sensitivity analysis was run on this parameter, meaning that in all 1000 simulations, this parameter value was sampled from a distribution. All other parameters were ranged by hand, generally tested on 1 simulation.

^b^ Range evaluated in deterministic sensitivity analyses.

For this analysis we added 2 health states to the model. First, we added the shelter no treatment state, which represents the shelter environment for individuals not taking MOUD or in detox. This health state is similar to the no treatment health state, but people in shelter no treatment have a higher probability of initiating buprenorphine in SBOT. People can transition to and from shelter no treatment and no treatment and to community treatment and incarceration (Figure 1). Second, we added shelter-based buprenorphine, which represents an SBOT program offering buprenorphine. People can transition to shelter-based buprenorphine from shelter no treatment, as well as from no treatment. Individuals who are enrolled in SBOT and who remain in care can transition to community-based buprenorphine (Figure 1). Individuals who discontinue SBOT care enter a 4-week posttreatment period with elevated overdose and mortality risk. We chose to model only shelter-based buprenorphine, and not shelter-based methadone, because federal regulations prevent prescription of methadone in outpatient settings such as SBOT. This strategy includes a case manager who helps with insurance enrollment, acquisition of identification documents, and other barriers to MOUD commonly faced by PEH.^7^

### Data and Parameter Estimation

The Massachusetts Public Health Data Warehouse (MA PHD) is the primary data source for demographics and treatment seeking parameters in RESPOND.^18^ MA PHD is a longitudinally linked administrative records database including service encounter data from over 16 Massachusetts agencies. MA PHD data inform demographics, overdose and all-cause mortality, and rates of receiving treatment. We utilized literature to inform OUD natural history and MOUD retention,^34,35,36^ federal supply schedule MOUD costs,^23,24^ and overdose costs.^24,37,38^ We used published estimates of health state utilities among persons who use drugs^39^ (see eAppendix 4 in Supplement 1 for details). Remaining parameters were calculated through empirical calibration. The process yielded 6000 parameter vectors simulating MA historical trends (eAppendix 5 in Supplement 1).^40^

For this study, we tailored a variety of parameters to simulate a population of PEH. (1) We increased nonoverdose mortality based on an analysis comparing mortality in unsheltered vs sheltered PEH and with the general Massachusetts population.^19^ (2) We increased the proportion of overdoses that were fatal by 2.3 based on a rate ratio from a study^22^ comparing the overdose risk among PEH vs housed individuals (Table 1). (3) We adjusted MOUD treatment starts in the community downward by 50% and detox admissions 2.1-fold higher, based on a study^41^ of OUD treatment among people who are unhoused vs housed (Table 1).

To estimate the size of the population of PEH with OUD in Massachusetts, we used the 2019 Census Point-in-Time estimate of homelessness and an estimated proportion of PEH within Boston with OUD.^42,43^ We adjusted age and sex distributions of this population to more closely represent PEH with OUD (Table 1).^3^

We conducted cost-effectiveness and sector budget impact analyses from a health care sector perspective and a modified societal perspective (which included incarceration costs). We denominated incarceration and health care costs in 2023 US dollars and discounted 3% annually.^44^ Budget impact analysis results were undiscounted. For treatment utilization costs for the shelter-based buprenorphine health state, we used costs from the community buprenorphine health state and added an estimate of the weekly per patient cost of a case manager (Table 1).^21,45^

### Statistical Analysis

First, we employed RESPOND to model a closed cohort of PEH with OUD residing in a Massachusetts shelter at the start of the simulation in 2013. This is similar to a cohort study in which every participant is exposed to the intervention and the model follows their progress over a lifetime. Next, we simulated over 10 years, starting in 2013, the total population of PEH with OUD in Massachusetts as an open cohort in which the PEH population members vary over time. This analysis includes all PEH, some who access emergency shelter and are thus exposed to the intervention, and some who do not. Over this period, the number of PEH was relatively stable.^44^ To enter SBOT, PEH who did not initiate the simulation residing in a shelter first had to transition into a shelter; thus, not every member of the population is exposed to the option of SBOT because not all PEH in the simulation transition to a shelter. This analysis simulates outcomes at the state-level if Massachusetts were to broadly implement SBOT. We performed probabilistic sensitivity analyses by running each strategy 1000 times, each time utilizing a different set of parameter values randomly selected from the underlying, multivariate, empirical distribution of the calibrated parameters (eAppendix 5 in Supplement 1). We summarized the results using aggregate resulted estimates and uncertainty intervals (UIs) which capture the parameter uncertainty of the RESPOND model. We conducted all analyses between the January 2023 and the April 2024 using R version 4.0.5 (R Project for Statistical Computing).

For both the open and closed cohort approaches, we modeled 2 strategies. (1) In status quo (SQ), the cohort progressed without the option to initiate buprenorphine in SBOT. (2) In shelter-based buprenorphine, the cohort progressed with the option to initiate buprenorphine in SBOT. We estimated that 65.2% of PEH with OUD who entered a shelter would initiate buprenorphine at the shelter clinic, based on initiation rates for buprenorphine from Boston Healthcare for the Homeless Program.^10^ We set the mean length of stay in SBOT to 4 weeks. We calculated linkage rates to community buprenorphine from shelter buprenorphine and withdrawal rates from shelter buprenorphine based on an 87% monthly linkage probability and a 42% monthly withdrawal probability from the literature.^21^ Table 1 presents the monthly transition rates (per 1000 people) that we calculated.

#### Outcomes

Clinical and public health outcomes included estimates of buprenorphine initiations, time spent taking MOUD, fatal overdose rates, life expectancy, and quality-adjusted life expectancy. Economic outcomes included health sector and modified societal perspectives costs, the components of health care spending, incremental cost-effectiveness ratios (ICERs) for the closed cohort, and budget impact for the open cohort. We calculated ICERs by dividing the incremental mean discounted lifetime cost between the 2 strategies by the incremental discounted quality-adjusted life-years (QALYs) gained. We considered a strategy as dominated when it was both more costly and yielded fewer QALYs than the alternatives.

#### Sensitivity Analyses

We performed a series of deterministic sensitivity analyses, varying key parameters one at a time to the upper and lower bounds of feasible ranges to identify influential variables and determine threshold values that could result in different conclusions. We varied 6 project-specific parameters: (1) linkage to community buprenorphine from shelter buprenorphine, (2) discontinuation of shelter buprenorphine, (3) initiation into shelter buprenorphine from shelter no treatment, (4) initiation into shelter buprenorphine from no treatment, (5) cost of a shelter-based buprenorphine program, and (6) standardized mortality ratios of PEH (eAppendix 5 in Supplement 1). We also varied several model parameters governing transitions in the community, including transition rates to office-based MOUD and overdose-related parameters. To visualize outcomes from deterministic sensitivity analyses, we utilized net monetary benefit with a willingness-to-pay threshold of $100 000 per QALY.^45^

## Results

### Closed Cohort of PEH With OUD

In the SQ scenario of the closed cohort of 13 800 PEH (mean [SD] age, 40.4 [13.1] years; 8749 male [63.4%]), discounted life expectancy was a mean (range) 22.00 (21.96-22.04) years and discounted, quality-adjusted life expectancy was a mean (range) of 4.24 (4.23-4.25) QALYs. The large difference in life expectancy and QALYs reflects discounting and low health state utilities for people actively using drugs and experiencing homelessness. The mean time spent on buprenorphine was 39.09 person-weeks (95% UI, 38.90-39.28 person-weeks) over an individual’s lifespan (Table 2). Without shelter-based buprenorphine, 37.1% (95% UI, 37.0%-37.2%) of the cohort ultimately died of an overdose. The mean (range) discounted lifetime cost per person was $123 978 ($123 931-$124 125) from the health sector perspective and $126 560 ($126 509-$126 712) from the modified societal perspective, with the $2582 per-person difference accounted for by incarceration costs.

**Table 2.** Health and Economic Outcomes of Shelter-Based Buprenorphine vs Status Quo

Outcome	Status quo, mean value (95% UI)	Shelter buprenorphine, mean value (95% UI)
Cohort of PEH with OUD over a lifetime		
Fatal overdoses (per person), No.	0.371 (0.370-0.372)	0.331 (0.330-0.333)
Person-weeks taking buprenorphine (per person), No.	39.09 (38.90-39.28)	92.99 (92.53-93.45)
Person-weeks taking shelter buprenorphine (per person), No.	0 (NA)	11.54 (11.48-11.60)
Total person-weeks taking buprenorphine (per person), No.	39.09 (38.90-39.28)	104.53 (104.01-105.05)
Costs per person (health care perspective), $	123 978 (123 931-124 125)	122 662 (122 506-122 819)
Incarceration costs per person, $	2582 (2578-2587)	2258 (2254-2261)
Costs per person (modified societal perspective), $	126 560 (126 509-126 712)	124 921 (124 760-125 080)
QALYs per person, No.	4.24 (4.23-4.25)	4.44 (4.43-4.45)
Life-years per person, No.	22.00 (21.96-22.04)	22.94 (22.90-22.98)
ICER (both cost perspectives), $ per QALY gained	NA	Dominant^a^
Population of PEH with OUD in MA over 10 y		
Fatal overdoses, No.	2756 (2743-2770)	2502 (2489-2514)
Person-weeks taking buprenorphine, No.	363 348 (361 213-365 483)	690 773 (686 668-694 878)
Person-weeks taking shelter buprenorphine, No.	0 (NA)	88 552 (88 011-89 092)
Total person-weeks taking buprenorphine, No.	363 348 (361 213-365 483)	779 325 (774 679-783 970)
Healthcare costs, $	3 069 578 882 (3 065 412 708-3 073 745 055)	3 003 191 912 (2 999 500 631-3 006 883 194)
Overdose costs, $	33 245 213 (33 084 412-33 406 014)	30 234 343 (30 085 345-30 383 340)
Pharmaceutical costs, $	30 770 274 (30 588 607-30 951 942)	54 533 396 (54 208 056-54 858 735)
Treatment costs, $^b^	234 291 296 (232 885 650-235 696 942)	258 148 555 (256 572 185-259 724 925)
Total costs (health care perspective), $	3 367 885 665 (3 362 155 090-3 373 616 239)	3 346 108 206 (3 340 539 112-3 351 677 299)
Incarceration costs, $	73 276 397 (73 101 500-73 451 294)	67 870 319 (67 730 819-68 009 819)
Total costs (modified societal perspective), $	3 441 162 062 (3 435 256 590-3 447 067 533)	3 413 978 524 (3 408 269 931-3 419 687 119)

Abbreviations: ICER, incremental cost-effectiveness ratio; MA, Massachusetts; NA, not applicable; OUD, opioid use disorder; PEH, people experiencing homelessness; QALY, quality-adjusted life-year; UI, uncertainty interval.

^a^ In our analysis, the intervention strategy of shelter-buprenorphine was dominant because there were lower costs and more QALYs gained in this strategy compared with status quo.

^b^ Treatment costs related to a visit to receive medications for OUD including physician and nursing time and tests, but not the costs of the medications.

In comparison, offering buprenorphine in SBOT was associated with a mean increase in life expectancy of 22.94 life-years (95% UI, 22.90-22.98), an additional 0.9 life-years per person compared with SQ, and discounted quality-adjusted life expectancy to a mean (range) 4.44 (4.43-4.45) QALYs, an additional 0.2 discounted QALYs per person compared with SQ. Offering buprenorphine in shelters was associated with an additional 65.4 person-weeks (167% increase) taking buprenorphine, with a mean of 11.54 person-weeks (95% UI, 11.48-11.60 person-weeks) taking buprenorphine in SBOT and a mean of 92.99 person-weeks (95% UI, 92.53-93.45 person-weeks) taking community buprenorphine over an individual’s lifespan (Table 2). We estimated that 33.1% of the population (95% UI, 33.0%-33.3%) would die of an overdose, an 11% relative reduction compared with SQ. The mean (range) discounted lifetime cost per person was $122 662 ($122 506-$122 819) from the health sector perspective and $124 921 ($124 760-$125 080) from the modified societal perspective with a mean (range) $2258 ($2254-$2261) per person in incarceration costs. The SBOT strategy was associated with greater discounted quality-adjusted life expectancy at a lower lifetime cost than the SQ from both health care ($1300 in savings per person) and modified societal cost perspectives, meaning that the SQ strategy was dominated (Table 2).

### Population Simulation of PEH With OUD in Massachusetts

**Figure 2.**
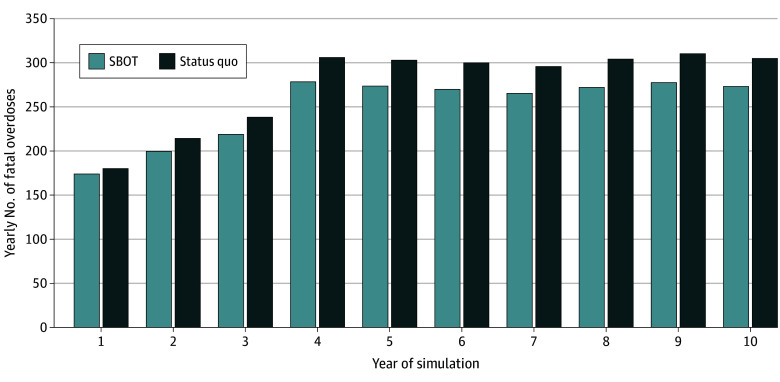
Yearly Fatal Overdoses in a Population Simulation of People Experiencing Homelessness (PEH) With Opioid Use Disorder (OUD) in Massachusetts This figure presents yearly fatal overdoses among PEH under status quo treatment availability and a shelter-based opioid treatment (SBOT) scenario, where shelters are an opportunity to initiate buprenorphine for treatment of OUD.

In the simulation of the entire population of PEH with OUD in Massachusetts, there were an additional 416 100 person-weeks taking buprenorphine (114% increase) with a mean 88 552 person-weeks (95% UI, 88 011-89 092 person-weeks) taking buprenorphine in SBOT (Table 2). Under the SQ strategy, we estimated a mean 2756 (95% UI, 2743-2770) overdose deaths at 10 years. In the shelter buprenorphine strategy, 254 overdose deaths were averted over the 10-year period, a 9.2% reduction in total overdose deaths among PEH in Massachusetts (Table 2 and Figure 2).

**Figure 3.**
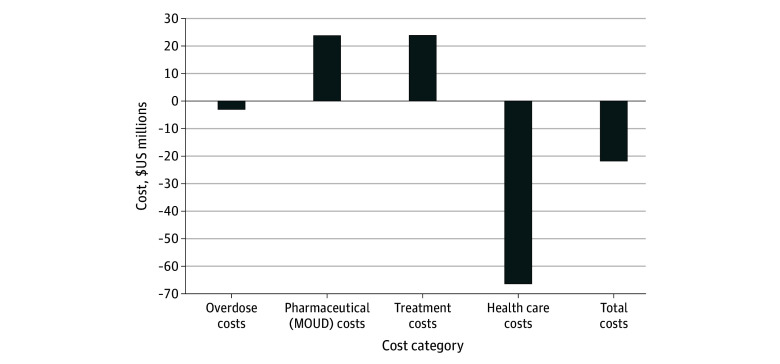
Total Discounted Cost Difference Between Shelter-Based Opioid Treatment (SBOT) Compared With Status Quo by Cost Category Cost differences on a 10-year time horizon (where negative costs involve cost savings) comparing status quo treatment availability and an SBOT scenario, where shelters are an opportunity for people experiencing homelessness to initiate buprenorphine for treatment of opioid use disorder. MOUD indicates medications for opioid use disorder.

In the SQ simulation, the projected 10-year undiscounted impact to the Massachusetts health care system was $3.368 billion (95% UI, $3.362 billion to $3.374 billion). From the modified societal perspective, there were $73.3 million (95% UI, $73.1 million to $73.5 million) in incarceration costs for a combined cost of $3.441 billion (95% UI, $3.435 billion to $3.447 billion) (Table 2). The impact of the shelter buprenorphine strategy was $3.346 billion (95% UI, $3.341 billion to $3.352 billion) from the health care sector perspective and $3.414 billion (95% UI, $3.408 billion to $3.420 billion) from the modified societal perspective, with $67.9 million (95% UI, $67.7 million to $68.0 million) in incarceration costs representing cost savings of $21.8 million in health care costs and $5.4 million in incarceration costs over 10 years (Table 2). The SBOT strategy generated $23.8 million in additional spending on MOUD pharmaceuticals and $23.9 million in additional spending on managing patients taking MOUD, but also substantial savings in spending to manage overdose ($3.0 million) and in all other health care utilization ($66.4 million) that more than offset the cost of SBOT to the health care sector (Table 2 and Figure 3). Overall, SBOT generated $44.7 million in additional medication and clinical costs, but saved $69.4 million in overdose and other health costs.

### Sensitivity Analyses

In deterministic sensitivity analyses, shelter-based buprenorphine continued to be cost saving in most analyses (eAppendix 5 in Supplement 1). Cost-effective, but not cost-saving, results occurred when we increased the base case shelter-based buprenorphine cost by more than 700%, decreased standardized mortality ratios for PEH with OUD by 40% or more, decreased the overdose rate by 50%, or decreased the proportion of overdoses that were fatal by 50%. The strategy had a cost-effectiveness ratio of more than $100 000 per QALY when we increased health care utilization costs by 50% or when community buprenorphine linkage from shelter-buprenorphine fell by 80.3 percentage points (to 17.8% per month from 98.1% per month) and the cost of shelter buprenorphine rose by 5000% (to $23 690 per person per month from $433 per person per month). In probabilistic sensitivity analyses, shelter-based buprenorphine was the preferred strategy in 100% of simulations (1000 of 1000 simulations) at a threshold of $100 000 per QALY (eAppendix 5 in Supplement 1).

## Discussion

In this economic evaluation study of clinical and economic outcomes of implementing SBOT across homeless shelters in Massachusetts, an SBOT strategy was associated with 9.2% fewer overdose deaths among PEH while being cost saving from health care sector and modified societal perspectives. The additional costs of pharmaceutical and associated patient-management costs of incorporating shelter-based buprenorphine visits were offset by decreased costs related to averted incarceration, overdose, and death. Although the estimated overdose rates (37.1% in SQ and 33.1% in the intervention) were high, Fine et al^3^ found that 1 in 3 patient deaths at Boston Healthcare for the Homeless were due to an overdose between 2014 and 2018 as synthetic opioid use became more common (Danielle Fine, MD, Department of Internal Medicine, Massachusetts General Hospital, personal communication).^46^ Expanding shelter-based buprenorphine in Massachusetts may be an effective strategy for addressing overdose deaths in a historically marginalized population.

This simulation modeling study extends prior work showing the feasibility of shelter-based buprenorphine programs^15^ and the effectiveness of buprenorphine programs tailored to PEH.^10^ Additionally, it extends prior findings using the RESPOND model, demonstrating the importance of high-yield settings for buprenorphine initiation. Prior studies have demonstrated that initiating buprenorphine at syringe-service programs^14^ and in correctional settings^47^ is cost-effective and lifesaving. Our findings reinforce the concept of touchpoints, including release from incarceration and clinic visits for injection-related infections—and now, homeless shelters—when starting MOUD could save lives and be potentially cost saving.^48^ Moreover, shelter-based buprenorphine is likely an equity-promoting tool. Overdose is the leading cause of death among PEH,^49^ who are known to die much earlier than their housed counterparts.^50^ Rates of overdose deaths in Massachusetts are highest among American Indian and Alaska Native, Hispanic, and Black individuals,^18^ who are disproportionately represented among PEH.^51^ Furthermore, it is known that structural racism shapes access to MOUD; for example, whether an individual has access to buprenorphine or methadone varies based on neighborhood segregation.^52^ Until permanent, supportive housing programs^53^ are broadly available, expanding shelter-based buprenorphine can address racialized inequities in MOUD access and, ultimately, outcomes.

The finding that shelter-based buprenorphine both averted overdose deaths and was cost saving was unexpected because interventions that extend life generally lead to higher spending. To confirm that shelter-based buprenorphine in Massachusetts would be cost saving, we conducted several sensitivity analyses where shelter-based buprenorphine continued to be cost-effective in almost every analysis. Of note, in accordance with guidelines for cost-effectiveness analyses,^44^ our analyses exclude start-up costs. Our model assumed the existence of shelter-based health care—in the style of Health Care for the Homeless programs—prior to rollout of shelter-based buprenorphine.^54^ Policymakers should take into account cost-effectiveness findings as well as start-up costs, and the budgetary impact of costs vs savings on different elements of the health care sector (eg, costs may be incurred by health care for the homeless organizations but savings may be achieved by hospitals). However, the prospect of a lifesaving, cost-saving intervention in the context of the overdose crisis is highly promising and deserves further study.

### Strengths and Limitations

This study has several strengths including the use of Massachusetts-specific data on individuals with OUD and parameters informed by studies of MOUD among PEH. This analysis is also subject to several limitations. First, factors affecting overdose are complex, and a simulation model cannot capture all such elements. Similarly, some model parameters remain uncertain, or are likely not accurate for every context. For example, linkage rates from shelter-based buprenorphine to community buprenorphine were important factors for cost-effectiveness but have not been definitively documented. While uncertainty in some parameters remains, we performed extensive sensitivity analyses to account for some key parameters when the direction of the uncertainty was unclear. While absolute projections of deaths are uncertain, the relative costs and benefits of the strategies are likely robust. Therefore, the policy conclusions of this analysis—that buprenorphine in shelter-based settings would save lives and provide value for cost (and may even be cost saving)—are likely robust. In addition, we simulated the impact of this intervention in Massachusetts, and overdose and cost projections may not generalize elsewhere. Additionally, our simulation ran from 10 years starting in 2013, which is the first year that data from the MA PHD were available. Data are not available beyond 2023. Whenever simulation modeling is used for projections (ie, projections beyond the data used for fitting and calibrating the model), results should be interpreted with caution because these results depend on the (sometimes strong) assumption that the trends observed would continue in a similar pattern. Furthermore, by definition, cost-effectiveness evaluations do not include start-up costs, although start-up costs could be a real-world barrier to starting an SBOT program. Boston Health Care for the Homeless Program, whose experience has shaped many of the assumptions in this model, was uniquely poised to shelter-based buprenorphine given existing shelter-based medical infrastructure and relationships with shelters. That said, in recent years, a variety of shelter- and street-based buprenorphine programs have emerged across the country out of a wide variety of organizations,^54,55,56^ indicating that SBOT is indeed likely to be feasible on a larger scale.

## Conclusions

The findings of this economic evaluation suggest that shelter-based buprenorphine distribution could markedly increase access to a lifesaving medication in a population that has historically faced substantial barriers to treatment. For state policymakers, implementing such an intervention statewide offers compelling benefits. Primarily, it has the potential to avert a substantial number of overdose deaths. Additionally, it could result in considerable cost savings, largely through reduced health care expenses. This approach not only addresses a critical public health issue but also presents a fiscally responsible solution.

## Notes

Supplement 1. **eAppendix 1.** Introduction **eAppendix 2.** Model Structure **eAppendix 3.** Model Data and Methodology **eAppendix 4.** Empirical Calibration **eAppendix 5.** Project-Specific Supplemental Results
eReferences

Supplement 2.
Data Sharing Statement
