# Supplementary material for: Health and Economic Outcomes of Offering Buprenorphine in Homeless Shelters in Massachusetts
Source: JAMA Netw Open. 2024 Oct 16;7(10):e2437233. doi: 10.1001/jamanetworkopen.2024.37233 (PMC11581564; doi:10.1001/jamanetworkopen.2024.37233)
Supplement: Supplement 1. — eAppendix 1. Introduction eAppendix 2. Model Structure eAppendix 3. Model Data and Methodology eAppendix 4. Empirical Calibration eAppendix 5. Project-Specific Supplemental Results eReferences [file jamanetwopen-e2437233-s001.pdf]

Supplemental Online Content

Chatterjee A, Stewart EA, Assoumou SA, et al. Health and economic outcomes of offering buprenorphine in homeless shelters in Massachusetts. *JAMA Netw. Open*. 2024;7(10);e2437233. doi:10.1001/jamanetworkopen.2024.37233

eAppendix 1. Introduction
eAppendix 2. Model Structure
eAppendix 3. Model Data and Methodology
eAppendix 4. Empirical Calibration
eAppendix 5. Project-Specific Supplemental Results
eReferences

This supplemental material has been provided by the authors to give readers additional information about their work.

© 2024 Chatterjee A et al. *JAMA Network Open.*

eAppendix 1. INTRODUCTION

The growing prevalence of Opioid Use Disorder (OUD) has resulted in an increase in opioid overdoses in the United States. Drug overdose is the leading cause of premature death among Americans under the age of 50 and has increased by more than 6 times between 1999 and 2021.[1] Although evidence-based treatments are available for treating OUD, these treatments are under-utilized, thus the impact of opioids on the United States' population persists.

Researchers and policy makers have made efforts to create feasible action plans for reducing the toll of overdose deaths. Unfortunately, most policy makers do not have the evidence needed for informing and implementing system-level change. System-level thinking investigates how systems operate and how they can be modified to produce desired outcomes. At this time, data on system-level interventions for OUD are limited and inconsistent.

In an effort to fill the knowledge gap, simulation modeling can be used to integrate data from multiple sources to translate outcomes from clinical studies to policy-relevant data about population health and cost. By simulating state-level behaviors and practices related to OUD, we can project and evaluate the impact of relevant interventions and policies on public health outcomes and costs, hence informing practice and policy decisions to combat OUD.

The **R**esearching **E**ffective **S**trategies to **P**revent **O**pioid **D**eath (RESPOND) model is a state-transition, cohort-based model that simulates populations with high-risk opioid use, including the natural history of OUD, movement on and from opioid treatment, and overdose. The model provides outputs and projections that decision-makers can use to evaluate and modify care delivery systems to match their local epidemics and available resources.

Model inputs and parameters are adaptable to users' needs, namely, to represent heterogeneous populations, different dynamics of the drug overdose epidemic, and the effectiveness of intervention strategies in the prevention of opioid-related harms. The user, for example, can customize among other things, the demographics, time in each cycle, transition probabilities between health statuses and treatment states, and the number of health states included in the model to represent different structures and disease dynamics of the underlying populations.

© 2024 Chatterjee A et al. *JAMA Network Open.*

eAppendix 2. Model Structure

RESPOND is a state-transition, cohort-based[2,3] model that simulates the population living within a jurisdiction and who have high-risk opioid use. Typically, RESPOND simulates the population of a state, but it can also simulate a smaller area, such as a town or rural community, depending on the model parameter values. The model employs a Markov process with a weekly cycle length to accurately reflect population dynamics, clinical progression, and treatment of OUD.

The model structure comprises four main components: 1) population dynamics, 2) natural history of OUD, 3) care delivery, and 4) mortality.

The population dynamics modules simulate the epidemiology and demography of the opioid epidemic. The user can create either an open or a closed cohort simulation. In an open cohort simulation, new population "arrives" to the simulation in every time step, such that the total population in the model reflects the size of the total population with OUD living in that jurisdiction. The arrival rate represents both the development of new OUD and migration into the state among those with existing opioid use. In a closed cohort, no cohort members enter the simulation and the size of the population in the simulation dwindles over time as cohort members die.

The core simulation (*Figure 1*) of the RESPOND model involves the simulation of the natural history of OUD as a relapsing and remitting disease over a lifetime. RESPOND simulates OUD as a series of transitions between four health states of opioid use: 1) active, non-injection, 2) non-active, non-injection, 3) active injection, and 4) non-active, injection opioid use. In each time-step of the simulation, population fractions move between opioid use states. The definitions of "active" and "injection" opioid use can vary (but must be pre-specified) depending on the users' needs and available information. In the RESPOND base case, "active" opioid use is defined as any reported use in the previous seven days. "Injection" opioid use reflects any injection in the preceding seven days (a person who is both injecting and using oral opioids would be categorized as "injection" in RESPOND).

The care delivery module (*Figure 2)* of RESPOND simulates OUD treatment and includes four treatment types: 1) outpatient buprenorphine (Bup), 2) outpatient injectable naltrexone (Ntx), 3) outpatient methadone maintenance (Mmt), and 4) inpatient acute drug detoxification (detox). The model is adaptable to additional intervention types to better reflect local conditions and evolutions in the treatment field. For this study, we added 3 blocks: 1) Corrections, 2) Shelter without buprenorphine treatment ("Shelter No Treatment"), and 3) Shelter-based buprenorphine treatment ("Shelter Buprenorphine"). In general, treatment episodes tend to decrease movement into active drug use, increase movement into non-active drug use, and have an independent effect on overdose rates

© 2024 Chatterjee A et al. *JAMA Network Open.*

conditional on active drug use. When population disengages from a treatment and is lost to follow-up, those people enter a corresponding “post-treatment state”. The post-treatment state is a fixed interval during which relapse to active drug use is high, tolerance to opioids is lower than before treatment, and the risk of drug overdose among those actively using opioids is higher than it is in the no treatment state. The post-treatment state represents the period of vulnerability and excess overdose observed in real-world settings among patients who have recently relapsed to opioid use after a period of sustained abstinence.

The mortality module simulates both overdose and competing risks of death. RESPOND simulates overdose mortality by first simulating overdose incidence as a function of age and type of drug use (injection vs. non-injection use). Next, the model simulates a probability of death conditional on having had an opioid overdose. The model simulates competing causes of death through the use of standardized mortality ratios that are a function of age, sex, and type of opioid use (injection vs. non-injection).

The primary model outputs are: 1) All-cause mortality, 2) Overdose mortality, and 3) Number of people on treatment.

The simulation process is as follows: At simulation start, the model initiates a cohort of people currently living with OUD in the jurisdiction of interest. Based on data from that jurisdiction, the model assigns the current population to a drug use state, as well as a treatment block, such that the simulated population, including the prevalence of OUD treatment, reflects the status quo. Moving forward through simulated time, the sequence of simulation steps are: 1) aging of the population, 2) arrival of new population, 3) transition between OUD drug use states, 4) transitions into and out of treatment, 5) overdose, and 6) death. At the end of this sequence of processes, the model advances simulated time by one cycle (week) and repeats the process. The simulation continues until a time horizon assigned by the user.

Full model schematics are available at https://www.syndemicslab.org/respond.

Following sections of this document provide details of model parameters for a specific use case of the model that simulates the OUD population of the state of Massachusetts from the end of year 2012-2015 with an open cohort.

Figure 1. Core Simulation

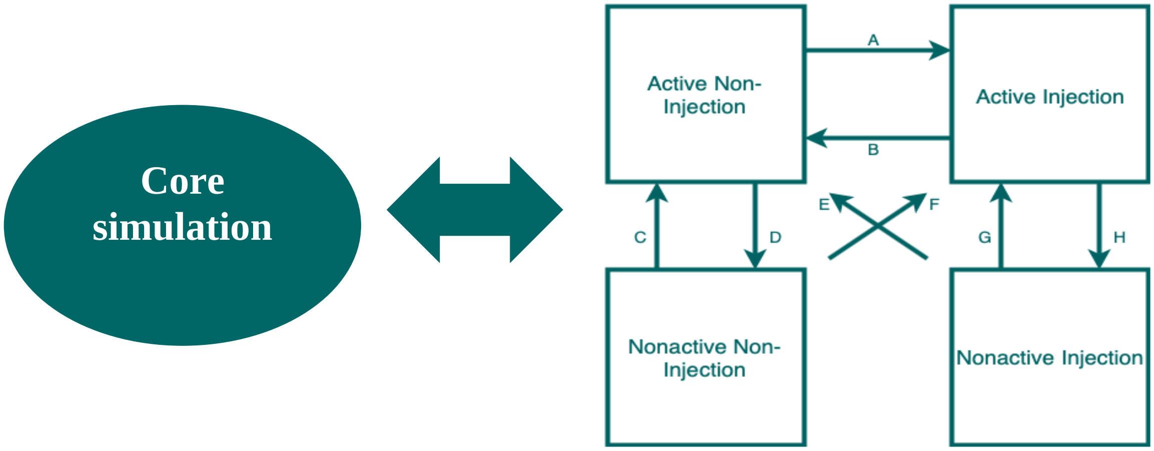

© 2024 Chatterjee A et al. *JAMA Network Open.*

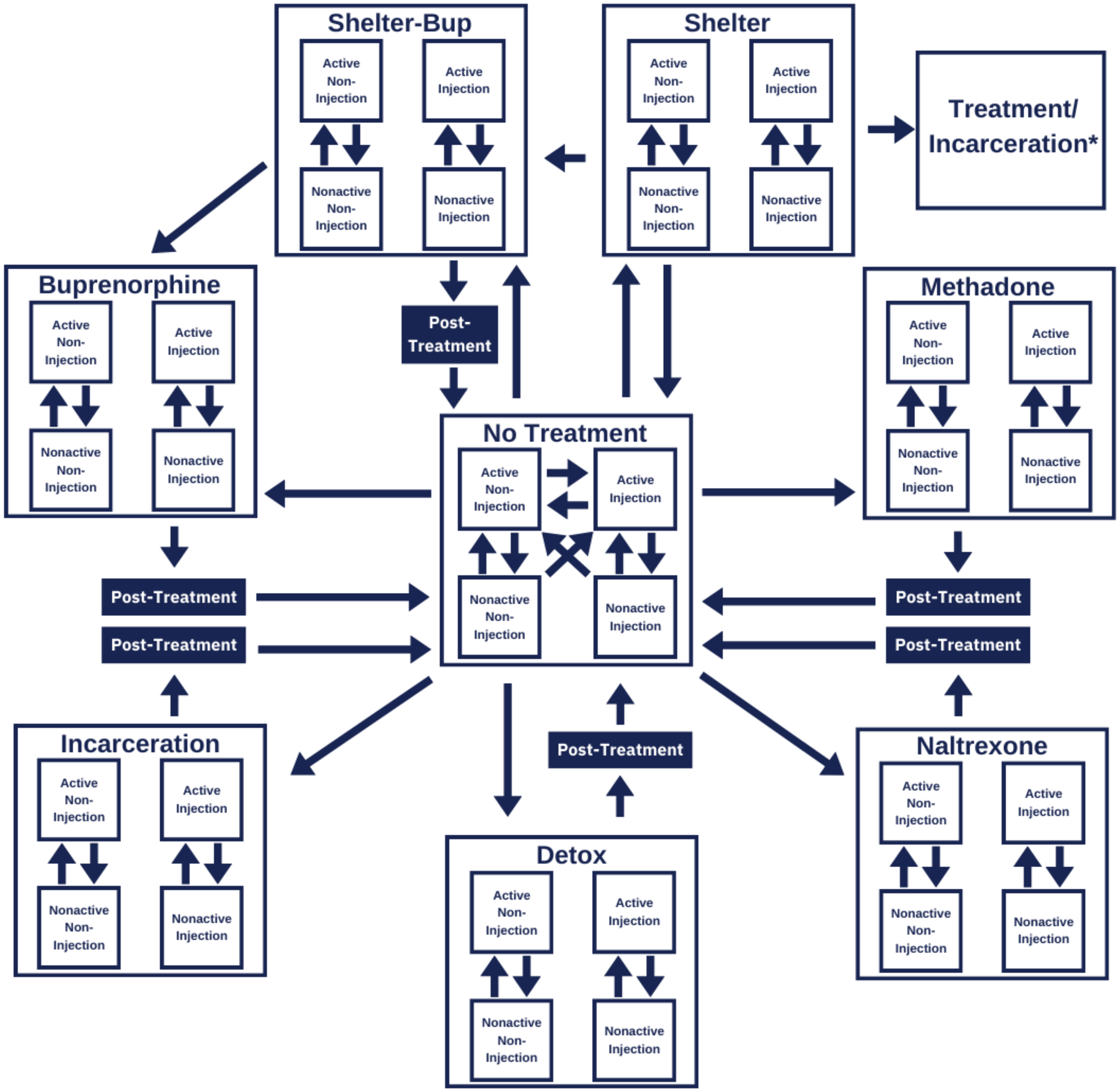

Figure 2. RESPOND's care delivery module with project specific health states

*People in the Shelter block can transition to all MOUD treatment, detoxification, and incarceration blocks.

© 2024 Chatterjee A et al. *JAMA Network Open.*

eAppendix 3. Model Data and Methodology

3.1.1 Massachusetts Public Health Data Repository

The Massachusetts Public Health Data Repository (MA PHD) is a linked longitudinal records dataset that includes administrative records and billing claims from over 15 state agencies.[4] The spine of the database is the Massachusetts All Payers Claims database, which includes medical billing for all payers in the state. The database links person-level records from vital statistics, the Department of Corrections, Emergency Medical Services, and the Bureau of Substance Addiction Services, such that it is possible to construct longitudinal, person-level trajectories across various treatment episodes, admissions to the hospital, and overdose events. RESPOND uses the MA PHD dataset to estimate parameters such as OUD epidemiology in MA and rates of transition onto treatments assuming the status quo.

3.1.2 NIDA Clinical Trial Network Protocols 0051 (CTN)

The National Institute on Drug Abuse administers a large clinical trials network for evaluation of treatments for substance use disorders. The Clinical Trial Network (CTN) 0051 protocol was a head-to-head comparative effectiveness trial of sub-lingual buprenorphine and injectable naltrexone for individuals with OUD who were accessing acute opioid detoxification services. RESPOND uses urine toxicology data from the trial to estimate bidirectional transitions between substance use states while taking buprenorphine or naltrexone, as well as health care utilization among patients with OUD.[5–7]

3.1.3 Medical Literature

In addition to the primary data sources listed above, RESPOND estimates many model parameters from the medical literature. The detailed explanation of model parameters below provides references to the relevant publications.

© 2024 Chatterjee A et al. *JAMA Network Open.*

3.2 Components

3.2.1 Population Dynamics

3.2.1.1 Initial Cohort

To simulate the demography and OUD epidemiology in the underlying population, RESPOND requires the initial cohort to be specified as follows: 1) age and sex distributions of people with OUD, 2) proportion of people beginning in each drug-use state, and 3) proportion of people within each treatment episode.

Structural Assumptions:

- No population begins the simulation in a post-treatment block.
- RESPOND does not characterize the population by race or ethnicity.

Methodological Notes:

Table 1 presents the key parameters related to cohort initialization.

For the base version of RESPOND, the following approach is taken to initialize the cohort. First, we took the estimates of the population of OUD between 2013 and 2015 from an age, sex, and county stratified capture-recapture analyses using the Massachusetts Public Health Data Warehouse (MA PHD).[8] The capture-recapture approach provides a method to estimate the total population with high-risk opioid use in a given calendar year, including those who have not been identified as being an opioid user and do not appear in medical claims or prevalence surveys. The previous work provides estimates for year 2012, however, as it has been noted in the paper, the data sources used before 2013 were not complete. Hence, we used data in the years with more comprehensive data sources to predict the data in 2012 which has fewer data sources.

Second, we obtained the age and sex stratified counts for alive OUD population at the end of each year by subtracting death with opioid overdose involved from the estimated total size.

Then, we applied a Negative Binomial regression on the alive counts to predict the counts in the end of 2012, with age, sex and calendar years being the covariates. An interaction of Age groups and sex was applied in the model. We treated this predicted count as the alive population size in the end of year 2012 and assumed that it constructs the initial cohort beginning in year 2013. To further identify the number of OUD in no treatment, as well as in treatment, at the beginning in year 2013, we identified OUD as we

© 2024 Chatterjee A et al. *JAMA Network Open.*

did in Barocas et al.[8] and then counted the number of OUD in treatment (N_0) in January 2013. The remaining population size is called "the number of OUD in no treatment"(T_0).

For this study, we utilized the base initial cohort as a starting point and adjusted the age and sex distributions to be more representative of the population of PEH in Massachusetts based on data from Fine et al.[9] For the population-level simulation, we distributed the cohort across OUD states based on the proportion of individuals in each state in RESPOND base case. To distribute the cohort across health states, we used the same distribution across treatment blocks as RESPOND base case and used the US Department of Housing and Urban Developments 2022 point-in-time estimate of the proportion of PEH who are sheltered[10] to distribute across shelter vs non-shelter states. We adjusted the cohort size based on our estimate that the population size of PEH in Massachusetts is approximately 13,800 based on data from the US Census and the proportion of PEH patients at Boston Healthcare for the Homeless with OUD.[10,11]

Table 1. Initializing Cohort Parameters

Parameter	Value	Method	Years	Stratification	Time Varying	Source
Population size, n	13,800	Estimated			No	[10,11]
Population of high-risk opioid use						
Total ($\hat{N}_{OUD,t}$)		Capture recapture	2013-2015	Age (3 groups*) & Sex	Yes	MA PHD analysis update to Barocas et al.[8]
By age-group		Observed	2012-2015	Age (18 groups**) & Sex	Yes	US Census 2010
Proportion with injection drug use	25.09%	Observed	2013	Age (3 groups*) & Sex	No	NSDUH
Proportion non-actively using	9%	Estimated			No	[12]

Abbreviations:
- MA PHD: Massachusetts Department of Public Health data
- NSDUH: National Survey on Drug Use and Health

* 3 age groups: 10 – 24, 25 – 44, 45 – 99
** 18 age groups: 5-year age-groups from 10-99

© 2024 Chatterjee A et al. *JAMA Network Open.*

3.2.1.2 Aging

RESPOND simulates discrete time steps (rather than continuous time) and categorical age groups or "brackets" over the lifetime. The user can define the bounds of age groups to match the structure of the underlying population. Aging occurs as the population progresses to the next age group after a number of cycles that is determined by the size of the age brackets. The model employs a half-cycle correction and aging occurs in discrete steps, namely only at multiples of the age group size.

Structural Assumptions:

- The entire population of the last age bracket (95 to 100-year-olds) is removed from the simulation at each aging cycle and replaced by the population from the previous age bracket.

3.2.1.3 Entering Cohort (New OUD Arrivals)

RESPOND can simulate either an open cohort (meaning that new individuals arrive to the population over the course of the simulation), or a closed cohort. Here we consider an open cohort simulation of OUD population in state of Massachusetts. When running the model to simulate an open cohort, arrivals to the population occur at every time step. Arrival rates are stratified by age and sex and vary over time to realistically reflect the incidence of new OUD and/or movement into the jurisdiction. For this study, we adjusted the size of the entering cohort to be approximately 6.5% of the RESPOND base case entering cohort, given that we estimated the population PEH with OUD is around 6.5% of the total population with OUD in MA.[10,11]

Structural Assumptions:

- All new populations enter the first block ("No Treatment" episode) and the first OUD state (currently active, non-injection).
- All new arrivals enter the simulation as active non-injectors under the "No Treatment" block but can transition to other feasible OUD states in the OUD transition module.

© 2024 Chatterjee A et al. *JAMA Network Open.*

Methodological Notes:

Table 2 and ***Table 3*** present the key parameters related to entering cohorts.

- The size of the entering cohort ($N_{enter,t}$) each year defined as:

$$N_{enter,t} = \hat{N}_{OUD,t} - (\hat{N}_{OUD,t-1} - \hat{N}_{FOD,t-1} - \hat{N}_{D\_other,t-1}) \qquad (1)$$

where $\hat{N}_{OUD,t}$ is the estimated OUD population in year t, $\hat{N}_{FOD,t-1}$ is the estimated number of fatal overdoses, and $\hat{N}_{D\_other,t}$ is the estimated total number of deaths from other causes, in the previous year (t-1).
- Let $\alpha_{j,k}(t)$ be age-sex stratified weekly new OUD arrival count in year t, where j, and k represent age and sex strata respectively and t=2013, 2014, 2015. Then $\alpha_{j,k}(\mathrm{t}) = \mathbf{A}(\mathbf{t}) \times p_{j,k}(\mathrm{t})$. Here $\mathbf{A}(\mathbf{t})$ is the weekly non-stratified total arrivals and $p_{j,k}(\mathrm{t})$ denotes yearly time varying demographic proportions. We calculate $\mathbf{A}(\mathbf{t})$ from $N_{enter,t}$ as $\mathbf{A}(\mathbf{t}) = N_{enter,t}/52$.

Table 2. Entering Cohort Parameters

**Parameter**	**Description**	**Value**	**Method**	**Stratification**	**Source**
$N_{initial}$	Initial cohort size combining no treatment size and treatment size	13,800	Estimated from US Census and BHCHP data	Age (j=1,..,3 groups*)<br>Sex (k=1,2)<br>Time (t=2013, 14, 15)	[10,11]
$N_{enter,t}$	Entering cohort size	$\hat{N}_{OUD,t} - \hat{N}_{initial,2013}$	Calculated	Time (t=2013)	MA PHD[4] [8]
$N_{enter,t}$		$\hat{N}_{OUD,t} - (\hat{N}_{OUD,t-1} - \hat{N}_{FOD,t-1} - \hat{N}_{D\_other,t-1})$	Calculated	Time (t=2014, 15)	
$\hat{N}_{FOD,t}$	Fatal overdoses		Estimated		
$\hat{N}_{D\_other,t}$	Non-FOD deaths		Estimated		MA PHD[4]
$p_{jk}(t)$	Entering cohort demographic proportions at each cycle (week)		- Observed Imputation of missing strata %	Age (j=1,..,5 groups**)<br>Sex (k=1,2)<br>Time (t=2013, 14, 15)	[13]

Abbreviations:
- MA DPH: Massachusetts Department of Public Health data
- NSDUH: National Survey on Drug Use and Health
- MRB: Methodological Resource Book
- NB: Negative Binomial Distribution

* 3 age groups: 10 – 24, 25 – 44, 45 – 99
** 5 age groups: 10 – 19, 20 – 24, 25 – 34, 35 – 49, 50 – 99
*** Other cause mortality is from Chapter 55 assuming that is no additional death in one year.

© 2024 Chatterjee A et al. *JAMA Network Open.*

Table 3. Entering Cohort Counts by Year

Total counts (population size)	Year			
	**2012*** **	**2013**	**2014**	**2015**
Population of OUD at time t ($\widehat{N}_{OUD,t}$) – RESPOND Base Case	166,835	226,861 (189832,301264)	233,184 (178671,271814)	275,070 (222160,357383)
Total number of non-FOD*deaths ($\widehat{N}_{D\_other,t}$) – RESPOND Base Case	596	964	1,332	1,605
Total number of FOD* deaths ($\widehat{N}_{FOD,t}$) – RESPOND Base Case	702	900	1,294	1,562
Entering Cohort (yearly total) ($N_{enter,t}$) – RESPOND Base Case	-	**10,943**	**8,187**	**44,512**
Entering Cohort (Weekly total) ($\mathbf{A}(\mathbf{t})$) – RESPOND Base Case	-	**210**	**157**	**856**
Entering Cohort (Weekly total) – Project Specific	-	**14**	**10**	**55**

*** The estimates and observed data in year 2012 do not include data from Emergency Medical Services (EMS). Subsequent years do include those data.

© 2024 Chatterjee A et al. *JAMA Network Open.*

3.2.2 Natural History of OUD

RESPOND simulates opioid use as a series of transitions through four opioid use health states: 1) Non-Active and 2) Active non-injection use, as well as 3) Non-Active and 4) Active injection use (*Figure 1*). Throughout the simulation, there is a multi-directional movement between OUD states.

Transitions between drug use compartments impact four important outcomes: 1) risk of overdose, 2) risk of death from competing causes, 3) health care utilization (cost), and 4) quality of life.

The primary sources of data for substance use transitions are studies from the medical literature.

Structural Assumptions:

- OUD is a remitting and relapsing process over a lifetime. There is no health state of OUD cure or permanent recovery.
- Transitions between OUD health states are not time updated.

Methodological Notes:

Table presents the key parameters related to OUD transitions for no treatment.

- All Confidence Intervals (CIs) are 95%, namely calculated at α=5% level of significance.
- CIs for proportions p_E, p_F, p_B, p_H, and p_D are calculated using the normal approximation to binomial proportions.
- CIs for rates R_A, R_C, and R_D are provided from the manuscript and calculated assuming Poisson distribution.
- Weekly rates and proportions, calculated from the respective overall estimates, are converted to weekly transition probabilities as indicated in the "Method" column in ***Table 4***.

© 2024 Chatterjee A et al. *JAMA Network Open.*

Table 4. No-Treatment and Post-Treatment: OUD Transition Parameters

Parameter	Description	Value	Method	Source
**No Treatment (ρ_N)**				
R_A	Rate of active non-injection to active injection	4.6 per 100 PY (3.0 , 6.6)		14
P_A	Probability of active non-injection to active injection	0.000884 (0.000577 , 0.001268)	Calculated from R_A: P_A= 1-exp{R_A/52}	
R_C	Rate of non-active non-injection to active non-injection	16 per 100 PY (12.0 , 20.5)		
R_G	Rate of non-active injection to active injection			
P_C	Probability of non-active non-injection to active non-injection	0.00307 (0.00230 , 0.00393)	Calculated from Rate(R_C): P= 1-exp{R_C/52}	
P_G	Probability of non-active injection to active injection			
p_B	Proportion of active injection to active non-injection	0.34		15
P_B	Probability of active injection to active non-injection	0.00067 (0.00054 , 0.0008)	Calculated from p_B: 1-exp(x) where x = ln(1-p_B)/(12*52)	
p_E	Proportion of non-active injection to active non-injection	0.13		
p_F	Proportion of non-active non-injection to active injection			
P_E	Probability of non-active injection to active non-injection	0.000223 (0.000115 , 0.00034)	Calculated from p_E: 1-exp(x) where x = ln(1-p_E)/(12*52)	
P_F	Probability of non-active non-injection to active injection			
p_D	Proportion of active non-injection to non-active non-injection	0.03 (0.0175 , 0.0425)		16
p_H	Proportion of active injection to non-active injection			
P_D	Probability of active non-injection to non-active non-injection	0.00058 (0.00032 , 0.00085)	Calculated from p_D: 1-exp(x) where x = ln(1-p_D)/(52)	
P_H	Probability of active injection to non-active injection			
**Post-Treatment (ρ_P)**				
P_A, P_B, P_D, P_E, P_F, P_H,	Same estimates with no-treatment.			
p^*_C	Proportion of non-active non-injection to active non-injection	0.65	CIs are calculated using the normal approximation to binomial proportions.	17
p^*_G	Proportion of non-active injection to active injection			
P_C	Probability of non-active non-injection to active non-injection	0.2308	Calculated from p: 1-exp(x) where x = ln(1-p)/4	
P_G	Probability of non-active injection to active injection			

© 2024 Chatterjee A et al. *JAMA Network Open.*

- p^*_C and p^*_G indicate the percentage of people relapsed within a month of discharge (after inpatient detoxification).
- The denominators for calculating weekly probabilities depend on whether the respective available proportion or rate estimates are yearly or monthly.

3.2.2.1 Health state utilities

We used published estimates of health state utilities among persons who use drugs. All quality-of-life measures are health state utilities collected using the standard gamble direct method of utility measurement.[18] We estimated multi-state utility functions using the minimal utility approach.[19] We employed the multiplicative utility approach in sensitivity analyses.

Table 5. Background Utilities

Age Group	Sex	Utility	Source
10_14	Male	0.922	[20]
10_14	Female	0.922	
15_19	Male	0.922	
15_19	Female	0.922	
20_24	Male	0.922	
20_24	Female	0.922	
25_29	Male	0.922	
25_29	Female	0.922	
30_34	Male	0.901	
30_34	Female	0.901	
35_39	Male	0.901	
35_39	Female	0.901	
40_44	Male	0.871	
40_44	Female	0.871	
45_49	Male	0.871	
45_49	Female	0.871	
50_54	Male	0.842	
50_54	Female	0.842	
55_59	Male	0.842	
55_59	Female	0.842	
60_64	Male	0.823	
60_64	Female	0.823	

© 2024 Chatterjee A et al. *JAMA Network Open.*

65_69	Male	0.823	
65_69	Female	0.823	
70_74	Male	0.79	
70_74	Female	0.79	
75_79	Male	0.79	
75_79	Female	0.79	
80_84	Male	0.763	
80_84	Female	0.763	
85_89	Male	0.763	
85_89	Female	0.763	
90_94	Male	0.763	
90_94	Female	0.763	
95_99	Male	0.763	
95_99	Female	0.763	

Table 6. Opioid Use State Utilities

Block	Opioid Use State	Utility	Source
No_Treatment	Active_Noninjection	0.626	[21]
No_Treatment	Active_Injection	0.512	
No_Treatment	Nonactive_Noninjection	1	
No_Treatment	Nonactive_Injection	1	
Buprenorphine	Active_Noninjection	0.71	
Buprenorphine	Active_Injection	0.71	
Buprenorphine	Nonactive_Noninjection	0.774	
Buprenorphine	Nonactive_Injection	0.774	
Naltrexone	Active_Noninjection	0.71	
Naltrexone	Active_Injection	0.71	
Naltrexone	Nonactive_Noninjection	0.774	
Naltrexone	Nonactive_Injection	0.774	
Methadone	Active_Noninjection	0.617	
Methadone	Active_Injection	0.617	
Methadone	Nonactive_Noninjection	0.758	
Methadone	Nonactive_Injection	0.758	

Detox	Active_Noninjection	1	
Detox	Active_Injection	1	
Detox	Nonactive_Noninjection	1	
Detox	Nonactive_Injection	1	
Post-Buprenorphine	Active_Noninjection	0.626	
Post-Buprenorphine	Active_Injection	0.512	
Post-Buprenorphine	Nonactive_Noninjection	1	
Post-Buprenorphine	Nonactive_Injection	1	
Post-Naltrexone	Active_Noninjection	0.626	
Post-Naltrexone	Active_Injection	0.512	
Post-Naltrexone	Nonactive_Noninjection	1	
Post-Naltrexone	Nonactive_Injection	1	
Post-Methadone	Active_Noninjection	0.626	
Post-Methadone	Active_Injection	0.512	
Post-Methadone	Nonactive_Noninjection	1	
Post-Methadone	Nonactive_Injection	1	
Post-Detox	Active_Noninjection	0.626	
Post-Detox	Active_Injection	0.512	
Post-Detox	Nonactive_Noninjection	1	
Post-Detox	Nonactive_Injection	1	

Table 7. Treatment State Utilities

**Block**	**Utility**	**Source**
No_Treatment	1	[22]
Buprenorphine	1	
Naltrexone	1	
Methadone	1	
Detox	0.78	
Post-Buprenorphine	1	
Post-Naltrexone	1	
Post-Methadone	1	
Post-Detox	1	

© 2024 Chatterjee A et al. *JAMA Network Open.*

3.2.2.2 Healthcare Utilization Costs

We calculated healthcare utilization costs based on data from Clinical Trial Network 0051[22,23] the provided 6 month costs in 2016 stratified by age, OUD status, and treatment block (No Treatment, Buprenorphine, and Naltrexone). We inflated these costs to 2023 values and divided to weekly costs. We set Methadone costs to be equal to Buprenorphine costs and Detox costs to 0, since Detox lasts one week and we assume no other OUD related healthcare utilization happens during that time. Post-treatment healthcare utilization costs was set to be equal to No Treatment costs (***Table 5***).

Table 8. Healthcare Utilization Costs

Block	Age	Sex	OUD Status	Healthcare Utilization Cost – Healthcare Perspective ($)
No Treatment/Shelter No Treatment/Post-Treatment	10-24	Male/Female	Active Non-injection	289
No Treatment/Shelter No Treatment/Post-Treatment	10-24	Male/Female	Active Injection	424
No Treatment/Shelter No Treatment/Post-Treatment	10-24	Male/Female	Non-active Non-injection	193
No Treatment/Shelter No Treatment/Post-Treatment	10-24	Male/Female	Non-active Injection	310
No Treatment/Shelter No Treatment/Post-Treatment	25-44	Male/Female	Active Non-injection	429
No Treatment/Shelter No Treatment/Post-Treatment	25-44	Male/Female	Active Injection	591
No Treatment/Shelter No Treatment/Post-Treatment	25-44	Male/Female	Non-active Non-injection	301
No Treatment/Shelter No Treatment/Post-Treatment	25-44	Male/Female	Non-active Injection	446
No Treatment/Shelter No Treatment/Post-Treatment	45-99	Male/Female	Active Non-injection	359
No Treatment/Shelter No Treatment/Post-Treatment	45-99	Male/Female	Active Injection	503
No Treatment/Shelter No Treatment/Post-Treatment	45-99	Male/Female	Non-active Non-injection	241
No Treatment/Shelter No Treatment/Post-Treatment	45-99	Male/Female	Non-active Injection	365
Buprenorphine/Shelter Buprenorphine/Methadone	10-24	Male/Female	Active Non-injection	193

© 2024 Chatterjee A et al. *JAMA Network Open.*

Buprenorphine/Shelter Buprenorphine/Methadone	10-24	Male/Female	Active Injection	305
Buprenorphine/Shelter Buprenorphine/Methadone	10-24	Male/Female	Non-active Non-injection	125
Buprenorphine/Shelter Buprenorphine/Methadone	10-24	Male/Female	Non-active Injection	223
Buprenorphine/Shelter Buprenorphine/Methadone	25-44	Male/Female	Active Non-injection	294
Buprenorphine/Shelter Buprenorphine/Methadone	25-44	Male/Female	Active Injection	430
Buprenorphine/Shelter Buprenorphine/Methadone	25-44	Male/Female	Non-active Non-injection	200
Buprenorphine/Shelter Buprenorphine/Methadone	25-44	Male/Female	Non-active Injection	320
Buprenorphine/Shelter Buprenorphine/Methadone	45-99	Male/Female	Active Non-injection	248
Buprenorphine/Shelter Buprenorphine/Methadone	45-99	Male/Female	Active Injection	372
Buprenorphine/Shelter Buprenorphine/Methadone	45-99	Male/Female	Non-active Non-injection	160
Buprenorphine/Shelter Buprenorphine/Methadone	45-99	Male/Female	Non-active Injection	267
Naltrexone	10-24	Male/Female	Active Non-injection	264
Naltrexone	10-24	Male/Female	Active Injection	385
Naltrexone	10-24	Male/Female	Non-active Non-injection	170
Naltrexone	10-24	Male/Female	Non-active Injection	274
Naltrexone	25-44	Male/Female	Active Non-injection	399
Naltrexone	25-44	Male/Female	Active Injection	548
Naltrexone	25-44	Male/Female	Non-active Non-injection	274
Naltrexone	25-44	Male/Female	Non-active Injection	331
Naltrexone	45-99	Male/Female	Active Non-injection	335
Naltrexone	45-99	Male/Female	Active Injection	466
Naltrexone	45-99	Male/Female	Non-active Non-injection	219
Naltrexone	45-99	Male/Female	Non-active Injection	331

© 2024 Chatterjee A et al. *JAMA Network Open.*

3.2.2.3 Cost of Shelter-Buprenorphine Health State

The cost of the shelter-buprenorphine health state combined the cost of the office-based buprenorphine health state and the cost of a case manager. We approximated the weekly, per patient cost of a case manager from the average of the U.S. median salaries of a Social and Community Service Manager and Social Worker from the Bureau of Labor Statistics in May 2022 and inflated to 2023 U.S. dollars.[24,25] We estimated patient capacity of a full-time case manager of 45 patients based on expert opinion **(*Table 6*)**.

Table 9. Cost of Shelter-Buprenorphine Health State

Parameter	Value	Source
Treatment Utilization Cost of Buprenorphine Blocks (2023)	$77.71	Physician Fee Schedule<br>Diagnostics Lab Fee Schedule
Social and Community Service Managers Median Salary in community and rehab services (2022 value inflated to 2023 USD)	$65,935	25
Social Worker Median Salary in mental health and substance abuse (2022 value inflated to 2023 USD)	$53,314	24
Full Time Case Manager Patient Capacity	45	Expert Opinion
Treatment Utilization Cost for Shelter Buprenorphine (2023)	$103.19	-

3.2.3 Care Delivery

RESPOND models OUD while engaged with treatment using the same 4-state opioid use simulation that it uses to model OUD without treatment. The 4-state OUD simulation is embedded within all treatment episodes (blocks), such that individuals may both remain engaged with treatment, but also experience periods of drug use relapse. Each treatment type has its own bi-directional transition probabilities between active and non-active use. The net movement between active and non-active use while engaged with treatment favors movement to non-active use over time.

RESPOND simulates treatment using the following parameters:

1. Probability of movement onto treatment from no treatment
2. Treatment initiation effect – the probability of ceasing active opioid use immediately after initiating treatment
3. Bi-directional movements between active and non-active opioid use while engaged with treatment

© 2024 Chatterjee A et al. *JAMA Network Open.*

4. Probability of loss to follow-up

The population that is lost to follow-up (disengages from care) must pass through a "post-treatment period" before rejoining the simulation of OUD. The post-treatment period is a four-week time, immediately following discontinuation of a treatment, during which the risk of relapse to drug use is high, as is the risk of overdose. Population that survives the post-treatment period transitions back to the simulation of OUD without treatment.

3.2.3.1 Movement From No-Treatment to Treatment Episodes

<u>Structural Assumptions:</u>

- Only population in active opioid use states seeks OUD treatment. Population that is not currently using opioids does not seek treatment.

The main source of data to inform the probability of transition from no treatment to a treatment episode for RESPOND base case is the MA PHD. For this study, we adjusted the RESPOND base case transition probabilities to reflect treatment seeking patterns among PEH.

<u>Methodological Notes:</u>

Let λ_{NT} denotes the weekly transition rates from no-treatment to treatment. Then, weekly transition probability from no-treatment to treatment is calculated from λ_{NT} as:

$$\widehat{\mathbf{P}}_{\mathbf{NoTrt} \rightarrow \mathbf{Trt.T}} = 1\text{- exp } \{-\lambda_{NT}\}$$

Where $\lambda_{NT} = \frac{\widehat{\mathbf{N}}_{\mathbf{Obs,NoTrt} \rightarrow \mathbf{Trt.T}}}{\widehat{\mathbf{N}}_{\mathbf{Total,NoTrt} \rightarrow \mathbf{Trt.T}}} \times \frac{1}{4}$.

Here $\widehat{\mathrm{N}}_{\mathrm{Obs,NoTrt} \rightarrow \mathrm{Trt.T}}$: the observed number of people with OUD who transitioned from no-treatment to treatment T in January 2013

$\widehat{\mathrm{N}}_{\mathrm{Total,NoTrt} \rightarrow \mathrm{Trt.T}}$: the total number of people with OUD "*at risk*" of transitioning from no-treatment to treatment T in January 2013

The weekly transition probability $\widehat{\mathrm{P}}_{\mathrm{NoTrt} \rightarrow \mathrm{Trt.T}}$ is estimated using data from the MA PHD repository[4], and is stratified by age (16 groups: 5-year age-groups from 10-85, and >85years old), sex, and treatment (T= Detox, Mmt, Ntx, and Bup).

© 2024 Chatterjee A et al. *JAMA Network Open.*

For this study, we calibrated RESPOND base case transition probabilities so that admissions to Detox were 2.1 times higher than base case values and admissions to office-based MOUD treatment were half as much as base case values based on data on treatment seeking patterns among PEH compared to the general population (***Table 10***).[26] Transition probabilities from No Treatment to Corrections come from previous work using the RESPOND model[27] and were calculated based on Massachusetts incarcerated population trends (***Table 11***).[28,29]

Table 10. Monthly Transition Rate (per 1,000 people) From No-Treatment to Treatment

	Closed Cohort			Open Cohort	
**Strategy**	**Mean Value**	**95% UI**	**Range Evaluated**	**Mean Value**	**95% UI**
**Buprenorphine**					
Status quo	8.630	8.617-8.643	4.315-12.945	9.029	9.016-9.042
Shelter Buprenorphine	3.981	3.975-3.987	1.990-5.971	3.848	3.842-3.854
**Naltrexone**					
Status quo	1.327	1.325-1.328	0.663-1.990	1.327	1.325-1.328
Shelter Buprenorphine	1.592	1.590-1.594	0.796-2.388	1.459	1.457-1.461
**Methadone**					
Status quo	2.653	2.650-2.657	1.327-3.980	2.653	2.650-2.657
Shelter Buprenorphine	3.317	3.312-3.322	1.659-4.976	2.653	2.650-2.657
**Detox**					
Status quo	33.964	33.914-34.015	16.982-50.947	40.792	40.731-40.853
Shelter Buprenorphine	40.792	40.731-40.853	20.396-61.188	44.210	44.144-44.276

*MOUD = Medications for Opioid Use Disorder

Table 11. Monthly Transition Rate from No Treatment to Corrections (per 1,000)

Age	Sex	OUD Status	Monthly Transition Rate to Corrections (per 1,000)
10-14	Male/Female	Active Non-injection + Injection<br>Non-active Non-injection + Injection	0
15-19	Male	Active Injection + Non-injection	4.642
15-19	Male	Non-active Injection + Non-injection	3.290
15-24	Female	Active Injection + Non-injection	0.754
15-24	Female	Non-active Injection + Non-injection	0.534

© 2024 Chatterjee A et al. *JAMA Network Open.*

20-24	Male	Active Injection + Non-injection	11.614
20-24	Male	Non-active Injection + Non-injection	8.231
25-34	Male	Active Injection + Non-injection	13.161
25-34	Male	Non-active Injection + Non-injection	4.594
25-34	Female	Active Injection + Non-injection	2.135
25-34	Female	Non-active Injection + Non-injection	0.746
35-44	Male	Active Injection + Non-injection	12.972
35-44	Male	Non-active Injection + Non-injection	4.011
35-44	Female	Active Injection + Non-injection	2.104
35-44	Female	Non-active Injection + Non-injection	0.651
45-54	Male	Active Injection + Non-injection	9.793
45-54	Male	Non-active Injection + Non-injection	6.120
45-54	Female	Active Injection + Non-injection	1.589
45-54	Female	Non-active Injection + Non-injection	0.994
55-99	Male	Active Injection + Non-injection	5.674
55-99	Male	Non-active Injection + Non-injection	3.788
55-99	Female	Active Injection + Non-injection	0.921
55-99	Female	Non-active Injection + Non-injection	0.615

3.2.3.2 Treatment Initiation Effect

When a population begins a treatment for OUD, for example out-patient buprenorphine, a portion of the population immediately transitions from active to non-active use. Following that initial “treatment initiation effect” there is bidirectional movement between active and nonactive use states, even while engaged with treatment. The main source of data for the treatment initiation effect and for substance use transitions while engaged with buprenorphine, naltrexone, or methadone is the NIDA CTN urine toxicology data. The CTN trials collected routine periodic urine toxicology from all participants. While the published clinical trials results censored participants at the first relapse to drug use (the primary outcome of that trial), the trials continued to collect data from patients who experienced a relapse, such that the database includes longitudinal urine toxicology from patients who relapsed to active use, as well as some who remitted back to non-active use over the course of the trial. We analyzed those data in an “as treated” manner, such that RESPOND estimates realistic movements between active and non-active drug use states among people who are taking a medication. Note that relapsing to active drug use is not the same thing as loss to follow-up from treatment (see below).

Methodological Notes:

Upon entering treatment, a proportion of the population immediately transitions from active to non-active opioid use. This proportion $\hat{p}_{Init\_Act \rightarrow NonAct}$ is stratified by treatment episode as follows:

- buprenorphine (Bup): 0.74, based on the proportion of observed negative (non-active) urine samples at week 1
- naltrexone (Ntx): 0.90, based on the proportion of observed negative urine samples at week 5
- methadone (Mmt): 0.57, based on the proportion of observed negative urine samples at week 5

We assume a binomial distribution and we use the Wald's method to calculate 95% CIs for the proportion $\hat{p}_{Act \rightarrow NonAct}$ representing the block initiation effect.

Table 12. Block Initiation Effects Parameters: Weekly Transition Probabilities Modeling Movement Between OUD States When Movement Between Treatment States Occurs

Initial OUD state	Transition to			
	Treatment (γ_T)			Post-treatment* (γ_P)
	Bup	Ntx	Mmt	
Active non-injection	0.257(0.204 , 0.309)	0.103(0.058 , 0.148)	0.433(0.403 , 0.462)	1
Active injection	0.257(0.204 , 0.309)	0.103(0.058 , 0.148)	0.433(0.403 , 0.462)	1
Non-active non-injection	N/A**	N/A**	N/A**	N/A ***
Non-active injection	N/A**	N/A**	N/A**	N/A ***

* These estimates are the same for Bup, Ntx, Mmt, and detox.

** There is no block initiation effect for population that is not currently using opioids, because only population that is currently using opioids seeks care in the model.

*** Calibration parameter.

3.2.3.3 Transitions Between Active and Non-Active Opioid Use While Engaged with Treatment

We estimated Weekly OUD transition probabilities $\hat{p}_{Trt\_Act \rightarrow NonAct}$ using Multi-State Models (MSMs).[30] We fit separate models for each treatment: buprenorphine (Bup), naltrexone (Ntx), and methadone (Mmt), using data from the National Institute of Drug Abuse Clinical Trials Network (NIDA CTN).[5–7]

© 2024 Chatterjee A et al. *JAMA Network Open.*

Structural Assumptions:

- Population engaged with treatment may move between active and non-active opioid use, but the population engaged with treatment does not change the route of administration of their opioid use. In other words, population that entered treatment using non-injection opioids will not escalate to injection drug use while still engaged with treatment (Core Simulation within OUD treatment episodes (blocks) – (*Figure*).

Transition probabilities between active and non-active states are the same for both injection and non-injection drug use. This structural assumption is confirmed by the MSM estimates for buprenorphine and methadone models, in which route was included as a model covariate, but was not a significant predictor of transition rates.

Methodological Notes:

- Each MSM includes age and sex as covariates.
- Age is included as a continuous covariate in the MSM model, thus allowing estimation of the transition probabilities for age bins in which data are not available. We consider five 5 age groups: 10–19, 20–24, 25–34, 35–49, and 50–99 years old.
- OUD transition for Buprenorphine and Methadone: We keep all the weekly MSM estimates of OUD transition probabilities except week 1, which is considered as block initiation.
- OUD transition for Naltrexone: We delete the estimates for the first 4 weeks due to the inaccurate results from detoxification. Week 5 is also excluded from the analysis, as it is considered as block initiation.
- Transition probabilities from non-active to active use are defined as: $\hat{p}_{Trt\_NonAct \rightarrow Act} = 1 - \hat{p}_{Trt\_Act \rightarrow NonAct}$
-

© 2024 Chatterjee A et al. *JAMA Network Open.*

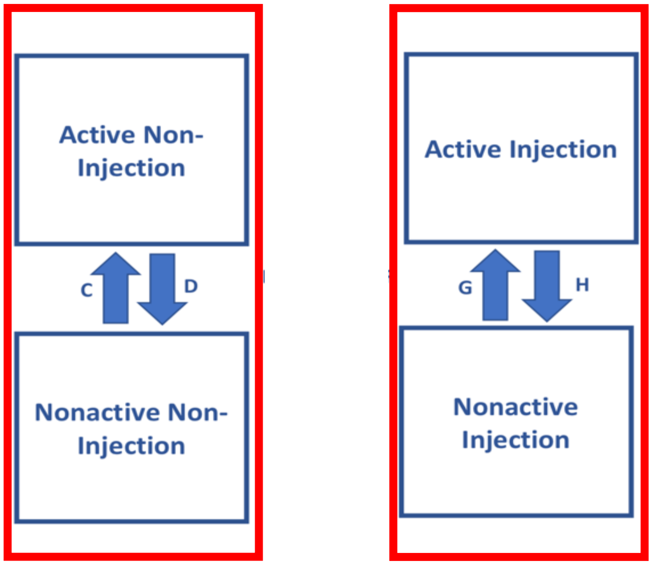

Figure 3. Core Simulation Within OUD Treatment Episodes (blocks)

Table 13. Transition Probabilities Between Active and Non-Active Opioid Use While Engaged with Treatment

Age	Sex	Initial OUD status*	Treatment		
			Buprenorphine	Naltrexone	Methadone
10-19	Male	Active	0.750	0.847	0.669
10-19	Female	Active	0.746	0.711	0.692
10-19	Male	Nonactive	0.156	0.107	0.112
10-19	Female	Nonactive	0.118	0.130	0.089
20-24	Male	Active	0.738	0.832	0.678
20-24	Female	Active	0.735	0.687	0.700
20-24	Male	Nonactive	0.148	0.099	0.120
20-24	Female	Nonactive	0.112	0.119	0.095
25-39	Male	Active	0.723	0.814	0.688
25-39	Female	Active	0.721	0.656	0.710
25-39	Male	Nonactive	0.139	0.090	0.128
25-39	Female	Nonactive	0.105	0.108	0.102
40-54	Male	Active	0.686	0.763	0.713
40-54	Female	Active	0.683	0.573	0.732
40-54	Male	Nonactive	0.119	0.072	0.152
40-54	Female	Nonactive	0.090	0.083	0.121
55-99	Male	Active	0.628	0.673	0.746
55-99	Female	Active	0.626	0.443	0.762
55-99	Male	Nonactive	0.096	0.051	0.192
55-99	Female	Nonactive	0.072	0.056	0.154

* Probabilities listed here are transition probabilities to "Active" state. For ex:- when initial OUD status is "Active", probabilities listed here are probabilities of staying in the "Active" state.

© 2024 Chatterjee A et al. *JAMA Network Open.*

3.2.3.4 Probability of Loss to Follow-Up

In every time step, the population that is engaged with treatment faces a risk of disengaging from care and being lost to follow-up. Loss to follow-up differs from relapse to active drug use while remaining engaged with opioid treatment. The population that disengages with care and is lost to follow-up enters the "post-treatment state," during which time they have a high rate of relapse to active use and a high rate of overdose among active users. The post-treatment block represents the period of time immediately following discontinuation of a medication or release from an abstinence-based setting (acute drug detoxification center, residential drug treatment, or jail), when opioid tolerance is low and the risk of overdose is higher than that of a person who never initiated treatment.

The main source of data for estimating the probability of loss to follow-up is Market Scan, a large insurance claims database containing millions of individuals who have commercial insurance coverage. As a randomized controlled trial, the CTN data cannot provide estimates of retention in care or loss to follow-up in the real world. We have previously published rates of loss to follow-up from buprenorphine and naltrexone treatment.[31] We therefore turn to Market Scan, which is nationally representative and reflects real-world practice in the U.S.

Structural Assumptions:

- In RESPOND, the only way to transition into a post-treatment episode is from a corresponding treatment episode.
- The "No Treatment" block does not have a post-treatment episode.
- RESPOND also considers the probability of immediate relapse to active opioid use upon being lost to follow-up from treatment:

Methodological Notes:

The weekly transition probability from treatment to post-treatment is calculated as[4]:

$$\widehat{\mathbf{P}}_{\mathbf{Trt} \rightarrow \mathbf{Post-Trt}\,.\mathbf{T}} = 1 - \exp\left(-\lambda_{TP}\right)$$

where

$\lambda_{TP} = \frac{-log\{1-(1-p_T)\}}{t}$ and p_T: the retention probability for treatment T reported for the study time-period t in literature.

© 2024 Chatterjee A et al. *JAMA Network Open.*

Table 14 presents estimates of the weekly transition probabilities $\widehat{P}_{Trt \rightarrow Post-Trt.T}$ based on data from Morgan et al.,[31] stratified by treatment.

Table 14. Weekly Transition Probabilities from Treatment *T* to Post-Treatment

**Treatment *T***	p_T	Study time-period t	λ_{TP}	$\widehat{\mathbf{P}}_{\mathbf{Trt \rightarrow Post-Trt.T}}$	**Source**
Bup	0.1760	52 weeks	0.0334	0.0328	31
Naltrexone	0.0214	52 weeks	0.0739	0.0713	
Methadone	0.5240	20 weeks	0.0323	0.0318	32

3.2.4 Overdose

Every person who is actively using opioids faces the risk of overdose. The probability of overdose depends on age, sex, and route of drug use (injection vs. non-injection). The simulation has no memory of past overdose events and does not include an elevated risk of repeat overdose after experiencing a first overdose event.

Structural Assumptions:

- Experiencing overdose has no independent impact on current or future opioid use behaviors.
- Only the population that is in an active opioid use state faces the risk of overdose.
- The risk of overdose is different between no-treatment, treatment, and post-treatment episodes.
- The risk of overdose is lower while engaged in treatment compared to not-engaged, even among the population who are actively using drugs while engaged with treatment.

Methodological Notes:

Counts of overdose are a target for model calibration. ***Table 15*** provides the empirically observed overdose fatalities from MA PHD.

© 2024 Chatterjee A et al. *JAMA Network Open.*

Table 15. Empirically Observed Opioid Overdoses in MA

Year	Age	Sex	Total number of people with opioid overdose	Number of fatal opioid overdose	Number of people with non-fatal overdose	Total overdoses
2013	10-19	Male	88	7	86	89
2013	10-19	Female	77	1	76	77
2013	20-24	Male	542	50	507	547
2013	20-24	Female	346	18	337	350
2013	25-39	Male	1929	264	1746	1966
2013	25-39	Female	975	99	899	995
2013	40-54	Male	1147	240	966	1178
2013	40-54	Female	776	121	673	791
2013	55+	Male	705	70	647	713
2013	55+	Female	630	30	596	631
2014	10-19	Male	112	13	107	113
2014	10-19	Female	101	1	101	101
2014	20-24	Male	823	75	778	833
2014	20-24	Female	502	33	482	508
2014	25-39	Male	3174	421	2898	3268
2014	25-39	Female	1415	141	1313	1443
2014	40-54	Male	1607	314	1368	1643
2014	40-54	Female	941	143	816	961
2014	55+	Male	913	105	825	928
2014	55+	Female	754	48	704	756
2015	10-19	Male	114	16	109	116
2015	10-19	Female	95	3	93	96
2015	20-24	Male	800	97	742	827
2015	20-24	Female	465	37	446	478
2015	25-39	Male	3804	545	3411	3914
2015	25-39	Female	1650	157	1537	1686
2015	40-54	Male	1863	357	1594	1919
2015	40-54	Female	973	142	871	1002
2015	55+	Male	1025	150	913	1045
2015	55+	Female	821	58	774	828

© 2024 Chatterjee A et al. *JAMA Network Open.*

3.2.4.1 Overall (non-block specific) overdose rates

Yearly rate $R_{OD,t}$ of overdose at time t for people not engaged in treatment is calculated as[5]:

$$R_{OD,t} = \frac{N_{OD,t}}{N_{OUD,t} + \frac{1}{2} \cdot N_{enter,t}} \times 1 \text{ PY}$$

for years t= 2013, 2014, 2015, assuming that each person contributes 1 person/year

where

$N_{OD,t}$: number overdose cases at time t

$N_{OUD,t}$: OUD cohort size at time t

$N_{enter,t}$: entering cohort size at time t

Therefore, weekly overall overdose rate from anywhere in the model $o(t) = {}^{R_{OD,t}}/_{52}$.

Table 16 presents point estimates of the overdose rates by age group, sex, OUD type, and year.

Table 16. Opioid Overdose Rates by Age, Sex, Year, and Type of OUD

Age	Sex	OUD	Years*		
			2013	2014	2015
10-19	Male	Active_Noninjection	0.000112076	0.000409791	0.000141081
10-19	Male	Active_Injection	0.000674857	0.002467526	0.000849508
10-19	Female	Active_Noninjection	0.000162217	0.000458584	0.000417609
10-19	Female	Active_Injection	0.000976778	0.002761329	0.002514598
20-24	Male	Active_Noninjection	0.001380621	0.000974182	0.000804356
20-24	Male	Active_Injection	0.008313304	0.005865957	0.004843369
20-24	Female	Active_Noninjection	0.001557061	0.001189503	0.000777667
20-24	Female	Active_Injection	0.00937572	0.007162497	0.004682664
25-39	Male	Active_Noninjection	0.000468337	0.000755758	0.00072912
25-39	Male	Active_Injection	0.002820052	0.004550736	0.004390337
25-39	Female	Active_Noninjection	0.00037227	0.000421042	0.000529921
25-39	Female	Active_Injection	0.002241592	0.002535274	0.003190876
40-54	Male	Active_Noninjection	0.000310914	0.000406988	0.000629183
40-54	Male	Active_Injection	0.001872141	0.002450645	0.003788574
40-54	Female	Active_Noninjection	0.000304432	0.000306566	0.000557584
40-54	Female	Active_Injection	0.001833113	0.001845965	0.003357447
55-99	Male	Active_Noninjection	0.000192418	0.000282608	0.000389897
55-99	Male	Active_Injection	0.001158631	0.0017017	0.002347731
55-99	Female	Active_Noninjection	0.00020927	0.000261909	0.000126691
55-99	Female	Active_Injection	0.0012601	0.001577063	0.000762859

* Overdose rates presented are weekly overall fixed rates o(t) for each year.

© 2024 Chatterjee A et al. *JAMA Network Open.*

Note that RESPOND model simulations has weekly time cycles. Let $\nu_B(\mathrm{t})$ be the overdose rate in a specific block where $B = N, T$ or P for blocks no-treatment, treatment, and post-treatment. Then, weekly overdose probabilities $P_{OD,B,t}$ are calculated from the respective overdose rates as[6]:

$$P_{OD,B,t} = 1 - e^{-\nu_B(t)}$$

where $B = N, T$ or P.

3.2.4.2 No Treatment

Weekly overdose rates in no treatment $\nu_N(\mathrm{t})$ were calculated by applying a multiplier $\mathbf{m_N} \in \mathbb{R}^+$ on overall overdose rates o(t) as $\nu_N(\mathrm{t}) = \mathrm{o(t)} \times \mathbf{m_N}$. There were no data available to inform the rate multiplier $\mathbf{m_N}$. Therefore, we decided to calibrate overdose rate multiplier of no treatment block.

3.2.4.3 Overdose While on Treatment

The risk of overdose for people engaged in treatment, is derived by applying a multiplier parameter $\mathbf{m_T}$ to the respective no-treatment $\nu_N(t)$ estimates. i.e., the weekly overdose rate at time t for treatment T is[7]:

$$\nu_T(\mathrm{t}) = \nu_N(t) \times \mathbf{m_T}$$

where $\nu_N(\mathrm{t})$ is the no-treatment overdose rate at time t, and $\mathbf{m_T} \in (0, 1)$.

Table 17. Multipliers of Overdose Rates by Treatment T

**Treatment** T*	$\mathbf{m_T}$	**95% CI**	**Source**
Buprenorphine	0.405	[0.35 , 0.46]	33
Naltrexone	0.864	[0.42, 1.31] injectable	
Methadone	0.752**	Non-parametric uncertainty distribution from bootstrapping of data	33,34

* We assume no overdoses occur in the detox block. Therefore, overdose rate is zero.
** $\mathbf{m_{Meth}} = \mathbf{m_{Bup}} \times \frac{\textbf{\textit{Meth rate}}}{\textbf{\textit{Bup rate}}} = 0.405 \times \frac{2.6}{1.4}$

© 2024 Chatterjee A et al. *JAMA Network Open.*

3.2.4.4 Overdose During the Post-Treatment Period

During the post-treatment period, individuals face a risk of overdose higher than that of people who never initiated a treatment. Therefore, we model post-treatment overdose rates $\nu_P(\mathrm{t})$ with a multiplier $\mathbf{m_P}$ (greater than 1) applied on no-treatment overdose rates such that $\nu_P(\mathrm{t}) = \nu_N(\mathrm{t}) \times \mathbf{m_P}$. There were no data available to inform post-treatment overdose rate multiplier $\mathbf{m_P}$. Therefore, we decided to calibrate overdose rate multiplier of post-treatments.

3.2.5 Mortality

RESPOND simulates mortality through two independent mechanisms, fatal opioid overdose and non-overdose death.

3.2.5.1 Fatal Overdose

The population that experiences overdose then faces a probability of death conditional on having had an overdose. This conditional probability of death, given an opioid overdose, is generalizable to all overdose cases and is therefore not stratified by age, sex, or OUD status. The population that survives an overdose does not change substance use as a result of the overdose. The probability of death conditional on having experienced an overdose is a time updated variable, reflecting changes to drug supply over time. Adjusting the conditional probability of overdose death provides a mechanism to reflect the growing penetration of fentanyl in local drug supplies, which is a major dynamic underlying mounting overdose deaths in the U.S. For this study, we maintained the same overdose rate, but increased the proportion of overdoses that were fatal by 2.3 based on a rate ratio from a study comparing drug overdose risk among PEH versus housed individuals.[35]

The probability $\mathbf{f}(\mathbf{t})$ of fatal overdose at year t is calculated as[8]:

$$\mathbf{f}(\mathbf{t}) = \frac{\mathrm{N_{FOD,t}}}{\mathrm{N_{OD,t}}} * 2.3$$

where $\mathrm{N_{FOD,t}}$ is the total number of fatal overdoses, and $\mathrm{N_{OD,t}}$ is the total number of all-type overdoses.

© 2024 Chatterjee A et al. *JAMA Network Open.*

**Table 18. Probabilities of fatal overdose.**	
**Year t**	$\mathbf{f(t)}$
2013	0.2870 (0.2670 , 0.3077)*
2014	0.2877 (0.2712 , 0.3045)*
2015	0.3096 (0.2933 , 0.3259)*
* Empiricaly calculated 95% CI using the Poisson distribution assumption for both counts in numerator and denominator of Eq. (7)	

3.2.5.2 Competing risks of death (non-overdose mortality)

Competing risks mortality includes deaths from conditions such as infectious endocarditis and sepsis, as well as medical comorbidities that accrue over a lifetime. The general approach to estimating competing risks of death is to apply standardized mortality ratios (SMRs) reflecting elevated mortality among drug users to age-sex stratified actuarial lifetables for the U.S.[36]

Table 19**Table** presents weekly death probabilities P_{NOD} calculated from lifetables in National Vital Statistic System[37] after excluding overdoses.

**Table 19. Overdose excluded weekly death probabilities derived from lifetables**		
**Age**	**Sex**	**Probability of Death**
10-14	Male	3.23e-06
10-14	Female	2.33e-06
15-19	Male	1.22e-05
15-19	Female	5.32e-06
20-24	Male	2.20e-05
20-24	Female	7.85e-06
25-29	Male	2.43e-05
25-29	Female	1.00e-05
30-34	Male	2.77e-05
30-34	Female	1.41e-05
35-39	Male	3.37e-05
35-39	Female	1.90e-05
40-44	Male	4.50e-05
40-44	Female	2.83e-05
45-49	Male	6.83e-05
45-49	Female	4.41e-05
50-54	Male	0.000113101
50-54	Female	7.06e-05
55-59	Male	0.000173492

55-59	Female	0.000105002
60-64	Male	0.000253182
60-64	Female	0.000149513
65-69	Male	0.000350328
65-69	Female	0.000223448
70-74	Male	0.000535512
70-74	Female	0.000363913
75-79	Male	0.00084698
75-79	Female	0.000601651
80-84	Male	0.001427294
80-84	Female	0.001055789
85-89	Male	0.002907422
85-89	Female	0.002393584
90-94	Male	0.002907422
90-94	Female	0.002393584
95-99	Male	0.002907422
95-99	Female	0.002393584

Weekly non-overdose death rates R_{NOD} are calculated as[38]:

$$R_{NOD} = \ln(1 - P_{NOD}) \times SMR$$

where SMR is calculated as[39]:

$$\text{SMR} = \frac{N_{D\_other}}{R_D \times N_{OUD}}$$

where N_{D_other} is the number of observed deaths not due to opioid overdose, R_D is the census death rate, and N_{OUD} is the size of the OUD population based on chapter 55 estimation.

We construct CIs around the SMR estimates assuming that the N_{D_other} follows a Poisson distribution and using the normal approximation (***Table 20***). For this study, we adjusted SMRs up by approximately 1.6 after comparing non-overdose related mortality among unsheltered PEH in the literature[40] to non-overdose related mortality in RESPOND base case.

**Table 20. Standardized Mortality Rates (SMRs)**		
**sex**	**OUD type**	**SMRs (95% CIs)**
Male	Active - Non-injection	2.85(2.52, 3.19)
Male	Active - Injection	7.03(6.12, 7.94)
Male	Non-active – Non-injection	2.91(1.83, 3.99)
Male	Non-active - Injection	7.31(4.32, 10.30)
Female	Active – Non-injection	3.65(3.17, 4.19)
Female	Active - Injection	9.04(7.67, 10.41)
Female	Non-active – Non-injection	3.67(2.06, 5.28)
Female	Non-active - Injection	8.96(4.57, 13.34)

Finally, we convert weekly non-overdose death rates to probabilities as $1 - e^{-R_{NOD}}$.

© 2024 Chatterjee A et al. *JAMA Network Open.*

3.2.5.3 Summary of the combined impact of medications for OUD on all-cause mortality in RESPOND

Medications for OUD (MOUD) have two independent effects on mortality that combine to provide synergies in the simulation:

1. The population that is engaged with MOUD treatment experiences a net movement toward non-active drug use. Because there is no risk of overdose while not using drugs, MOUD tend to decrease the rate of overdose in the population. In addition, movement out of active drug use states reduces exposure to the high standardized mortality ratios (SMRs) of active drug use and thereby reduce non-overdose mortality as well.
2. Among those who are actively using drugs when taking an MOUD, the MOUD has an independent effect on overdose risk, such that even those who are using have lower risk of death than those who are using drugs while not engaged with MOUD treatment

© 2024 Chatterjee A et al. *JAMA Network Open.*

eAppendix 4. Empirical Calibration

4.1.1 Calibration Summary

We used an empirical calibration approach to identify parameter vectors that provided accurate simulation of historical trends in MA overdose rates, the size of the population using opioids, and rates of admission to medically supervised opioid withdrawal (detox) services. The approach involves Latin hypercube sampling for searching a multidimensional parameter space comprising arrivals, overdose rates, treatment transition rates, and substance use state transition probabilities. The algorithm accepts proposed parameters when the respective model outputs lie within pre-determined target uncertainty ranges. This is an iterative process resulting in a set of parameter values for which the model hits all the calibration targets. The result of the calibration exercise was a set of approximately 6,000 parameter vectors that provided good fits to historical trends.

For project-specific Probability Sensitivity Analyses (PSAs), strategies are run on 1,000 of these parameter vectors, providing a range of output values representing an uncertainty interval. The PSA for this project was conducted based on results from the empirical calibration of the RESPOND model. The empirical calibration resulted in 6000 vectors of accepted values for the calibrated parameters, namely parameter values for which the RESPOND model simulated outcomes of interest close to the pre-specified calibration targets. These vectors represent the empirical multivariate distribution of the calibrated parameters. We did not specify univariate, marginal distributions (e.g., beta for model parameters representing probabilities, etc.). The multivariate, empirical distribution provides a more comprehensive representation of the underlying parameter uncertainty of the system that the RESPOND model tries to simulate, as it also accounts for the covariance/correlation between those calibrated parameters. By randomly drawing parameter values from this multivariate empirical distribution and running the RESPOND model multiple times (in our case 1000 times), we were able to capture in the respective UIs for the simulated outcomes of interest the parameter uncertainty inherent in the model.

4.1.2 Calibration Parameters

Table 21. List of Calibration Parameters of RESPOND

Model parameter	Stratification and time dependency	Data source	Calibration parameter?
1. Entering cohort counts<br>(3 different parameters for three years)	➢ Yearly time varying (i.e., considered fixed per cycle within a single year)	[8]	No
2. Entering cohort demographic proportions	➢ Age-gender stratified<br>➢ Yearly time varying (i.e.,	NSDUH dataset from 2013-2015	No

© 2024 Chatterjee A et al. *JAMA Network Open.*

(10 parameters each year = 30 altogether)		considered fixed per cycle within a single year)		
3. Block transition probabilities	1. Transitions from no-treatment to treatment blocks: Bup, Ntx, Meth and Detox (i.e., treatment initiation probabilities)<br>(16*2*4=128 parameters)	➢ Age-gender stratified<br>➢ Not stratified by route: injection vs non-injection for active users and non-active users are not allowed to transition to treatment blocks<br>➢ Stratified by treatment blocks<br>➢ No time variability	Chapter 55	No
	2. Transition probabilities for transitions from a treatment block to its post-treatment episode for Bup, Ntx, and Meth. For detox this probability is fixed at 1.<br>(3*2=6 parameters)	➢ Not age-gender stratified<br>➢ Stratified only by the active/non-active OUD status not by route<br>➢ Stratified by treatment blocks<br>➢ No time variability	[41]	No
	3. Transition probabilities for transitions from post-treatment episodes to no-treatment block<br>(1 parameter)	➢ Fixed value at 1/4	Expert opinion	No
4. Block initiation effects (probability of keeping the same OUD state when transitioning to a different block)<br>(3*2+4*2)=14 parameters		➢ Stratified by destination blocks (For Detox, fixed at zero, and no initiation effect in no-treatment block)<br>➢ When transitioning to MOUD blocks, non-active users keep the same OUD state with probability 1. When transitioning to post-treatment episodes, active users keep the same OUD state with probability 1<br>➢ Does not differ by the initial block that someone	Treatment initiation effects are estimated from CTN data.<br>[42]	Yes

		is transitioning from		
5. OUD transition probabilities	1. OUD transitions in no-treatment block and post-treatment episodes<br><br>80 parameters altogether	➢ Age stratified but not stratified by gender<br>➢ Stratified by all four OUD states (no transitions allowed between non-active states, from active non-injection to non-active injection, from active injection to non-active non-injection<br>➢ Not stratified by blocks for post-treatment episodes (i.e., the same values are used in all post-treatment episodes)	Several literature manuscripts.	Yes
	2. OUD transitions in treatment blocks: Bup, Ntx, and Meth (i.e., treatment efficacy). For Detox, we assume people keep their initial OUD status and therefore, fixed.<br><br>60 parameters	➢ Stratified by both age and gender<br>➢ Only stratified by active vs non-active OUD states without route<br>➢ Stratified by blocks and multi-state models are used to estimate the probabilities in each block of MOUDs	CTN data	No
6. All types overdose proportions	1. All types overdose proportions in no-treatment block and post-treatment episodes of Bup, Ntx, Meth and Detox<br><br>Altogether 60 parameters for three years only for no-treatment block + a multiplier.<br>For post-treatment episodes we have another 60 parameters	➢ Age-gender stratified<br>➢ Stratified by route: injection vs non-injection for active users<br>➢ Yearly time varying (i.e., considered fixed per cycle within a single year)<br>➢ Not stratified by blocks for post-treatment episodes (i.e., the same values are used in all post-treatment episodes)	Chapter 55	Yes
	2. All types overdose proportions in treatment blocks: Bup, Ntx, and Meth, and	➢ Calculated as a multiplier on the values of no-treatment blocks. Therefore, same stratification and time	Several literature manuscripts	Yes

© 2024 Chatterjee A et al. *JAMA Network Open.*

	zero for Detox<br>3 multipliers for each treatments	dependency as no-treatment block.		
7. Fatal overdose proportions (Calculated condition on overdoses)<br>Altogether 3 parameters for three years		➢ Yearly time varying (i.e., considered fixed per cycle within a single year)	Chapter 55	No
8. Standard Mortality Ratios (SMR)<br>8 parameters		➢ Stratified by gender and OUD status<br>➢ Not stratified by block. (i.e., same parameter values are used in all blocks)	8<br>4	No

© 2024 Chatterjee A et al. *JAMA Network Open.*

eAppendix 5. Project-Specific Supplemental Results

5.1.1 Deterministic Sensitivity Analyses

Table 22. One-Way Deterministic Sensitivity Analysis Results

Linkage to Community Buprenorphine from Shelter Buprenorphine				
**Strategy**	**Fatal Overdoses (per person)**	**Costs per Person ($)**	**QALYs per person**	**NMB (WTP of $100,000/QALY)**
**Decrease by 10%**	0.3270	121,032	4.4617	25,360
**Decrease by 20%**	0.3285	121,002	4.4560	24,819
**Decrease by 30%**	0.3298	120,977	4.4510	24,340
**Decrease by 40%**	0.3314	120,947	4.4452	23,795
**Decrease by 50%**	0.3327	120,920	4.4400	23,296
**Decrease by 60%**	0.3343	120,890	4.4342	22,747
**Decrease by 70%**	0.3357	120,862	4.4287	22,227
**Decrease by 80%**	0.3372	120,831	4.4228	21,674
**Decrease by 90%**	0.3387	120,802	4.4171	21,132
**Withdrawal from Shelter Buprenorphine**				
**Strategy**	**Fatal Overdoses (per person)**	**Costs per Person ($)**	**QALYs per person**	**NMB (WTP of $100,000/QALY)**
**Increase by 25%**	0.3269	121,023	4.4621	25,411
**Increase by 50%**	0.3280	120,991	4.4580	25,027
**Increase by 75%**	0.3290	120,964	4.4544	24,694
**Increase by 100%**	0.3298	120,940	4.4512	24,404
**Increase by 125%**	0.3306	120,919	4.4485	24,149
**Increase by 150%**	0.3312	120,900	4.4460	23,923
**Increase by 175%**	0.3318	120,883	4.4438	23,721
**Increase by 200%**	0.3323	120,868	4.4419	23,540
**Increase by 225%**	0.3328	120,854	4.4401	23,377
**Increase by 250%**	0.3333	120,842	4.4385	23,230
**Increase by 275%**	0.3337	120,830	4.4370	23,095
**Increase by 300%**	0.3340	120,819	4.4357	22,973
**Initiation of Shelter Buprenorphine from Shelter No Treatment**				
**Strategy**	**Fatal Overdoses (per person)**	**Costs per Person ($)**	**QALYs per person**	**NMB (WTP of $100,000/QALY)**
**Decrease by 10%**	0.3282	121,153	4.4556	24,631
**Decrease by 20%**	0.3309	121,250	4.4438	23,352
**Decrease by 30%**	0.3338	121,351	4.4315	22,021
**Decrease by 40%**	0.3367	121,456	4.4187	20,635
**Decrease by 50%**	0.3398	121,565	4.4053	19,190
**Decrease by 60%**	0.3431	121,679	4.3914	17,683

© 2024 Chatterjee A et al. *JAMA Network Open.*

**Decrease by 70%**	0.3464	121,797	4.3769	16,109
**Decrease by 80%**	0.3500	121,921	4.3616	14,464
**Decrease by 90%**	0.3537	122,050	4.3457	12,742
**Initiation of Shelter Buprenorphine from No Treatment**				
**Strategy**	**Fatal Overdoses (per person)**	**Costs per Person ($)**	**QALYs per person**	**NMB (WTP of $100,000/QALY)**
**Decrease by 10%**	0.3258	121,068	4.4659	25,738
**Decrease by 20%**	0.3261	121,077	4.4647	25,615
**Decrease by 30%**	0.3264	121,086	4.4636	25,491
**Decrease by 40%**	0.3266	121,095	4.4624	25,368
**Decrease by 50%**	0.3269	121,104	4.4613	25,243
**Decrease by 60%**	0.3272	121,113	4.4601	25,119
**Decrease by 70%**	0.3274	121,123	4.4590	24,994
**Decrease by 80%**	0.3277	121,132	4.4578	24,869
**Decrease by 90%**	0.3280	121,141	4.4566	24,743
**Cost of Shelter Buprenorphine**				
**Strategy**	**Fatal Overdoses (per person)**	**Costs per Person ($)**	**QALYs per person**	**NMB (WTP of $100,000/QALY)**
**Increase by 50%**	0.3256	121,269	4.4670	25,651
**Increase by 100%**	0.3256	121,478	4.4670	25,441
**Increase by 150%**	0.3256	121,688	4.4670	25,231
**Increase by 200%**	0.3256	121,898	4.4670	25,022
**Increase by 250%**	0.3256	122,108	4.4670	24,812
**Increase by 300%**	0.3256	122,318	4.4670	24,602
**Increase by 350%**	0.3256	122,527	4.4670	24,392
**Increase by 400%**	0.3256	122,737	4.4670	24,182
**Increase by 450%**	0.3256	122,947	4.4670	23,973
**Increase by 500%**	0.3256	123,157	4.4670	23,763
**Increase by 550%**	0.3256	123,367	4.4670	23,553
**Increase by 600%**	0.3256	123,576	4.4670	23,343
**Increase by 650%**	0.3256	123,786	4.4670	23,133
**Increase by 700%**	0.3256	123,996	4.4670	22,924
**Increase by 750%**	0.3256	124,206	4.4670	22,714
**Increase by 800%**	0.3256	124,416	4.4670	22,504
**Standardized Mortality Ratios**				
**Strategy**	**Fatal Overdoses (per person)**	**Costs per Person ($)**	**QALYs per person**	**NMB (WTP of $100,000/QALY)**
**Decrease by 10%**	0.3854	121,958	4.4989	28,150
**Decrease by 20%**	0.3715	122,901	4.5323	30,544
**Decrease by 30%**	0.3598	123,895	4.5673	33,061

© 2024 Chatterjee A et al. *JAMA Network Open.*

**Decrease by 40%**	0.3497	124,947	4.6044	35,717
**Decrease by 50%**	0.3408	126,068	4.6439	38,541
**Decrease by 60%**	0.3328	127,274	4.6862	41,567
**Transition to Buprenorphine from No Treatment/Shelter No Treatment**				
**Strategy**	**Fatal Overdoses (per person)**	**Costs per Person ($)**	**QALYs per person**	**NMB (WTP of $100,000/QALY)**
**Decrease by Half**	0.3280	121,125	4.4563	24,730
**Increase by Half**	0.3232	120,994	4.4774	26,962
**Transition to Naltrexone from No Treatment/Shelter No Treatment**				
**Strategy**	**Fatal Overdoses (per person)**	**Costs per Person ($)**	**QALYs per person**	**NMB (WTP of $100,000/QALY)**
**Decrease by Half**	0.3268	120,971	4.4617	25,417
**Increase by Half**	0.3244	121,145	4.4722	26,300
**Transition to Methadone from No Treatment/Shelter No Treatment**				
**Strategy**	**Fatal Overdoses (per person)**	**Costs per Person ($)**	**QALYs per person**	**NMB (WTP of $100,000/QALY)**
**Decrease by Half**	0.3280	121,146	4.4558	24,658
**Increase by Half**	0.3232	120,974	4.4778	27,031
**Transition to Detox from No Treatment/Shelter No Treatment**				
**Strategy**	**Fatal Overdoses (per person)**	**Costs per Person ($)**	**QALYs per person**	**NMB (WTP of $100,000/QALY)**
**Decrease by Half**	0.3385	121,063	4.3586	15,013
**Increase by Half**	0.3163	121,122	4.5497	34,071
**Overdose Rate**				
**Strategy**	**Fatal Overdoses (per person)**	**Costs per Person ($)**	**QALYs per person**	**NMB (WTP of $100,000/QALY)**
**Decrease by Half**	0.2022	127,079	4.6484	37,984
**Increase by Half**	0.4094	116,189	4.3165	15,680
**Fatal Overdose Proportion**				
**Strategy**	**Fatal Overdoses (per person)**	**Costs per Person ($)**	**QALYs per person**	**NMB (WTP of $100,000/QALY)**
**Decrease by Half**	0.2022	127,960	4.6484	37,103
**Increase by Half**	0.4094	115,467	4.3165	16,402
**Cost of Overdose**				
**Strategy**	**Fatal Overdoses (per person)**	**Costs per Person ($)**	**QALYs per person**	**NMB (WTP of $100,000/QALY)**
**Decrease by Half**	0.3256	120,474	4.4670	26,445
**Increase by Half**	0.3256	121,643	4.4670	25,276
**Cost of Healthcare Utilization**				
**Strategy**	**Fatal Overdoses (per person)**	**Costs per Person ($)**	**QALYs per person**	**NMB (WTP of $100,000/QALY)**
**Decrease by Half**	0.3256	66,570	4.4670	80,350

© 2024 Chatterjee A et al. *JAMA Network Open.*

**Increase by Half**	0.3256	175,547	4.4670	-28,628

Table 23. Two-Way Deterministic Sensitivity Analysis Results: Linkage to Community Buprenorphine from Shelter Buprenorphine by Cost of Shelter Buprenorphine

Linkage Strategy	Cost Strategy	Fatal Overdoses (per person)	Costs per Person ($)	QALYs per Person	NMB (WTP of $100,000/QALY)
Decrease by 10%	Increase by 100%	0.3270	121,454	4.4617	24,938
	Increase by 200%	0.3270	121,876	4.4617	24,516
	Increase by 300%	0.3270	122,298	4.4617	24,094
	Increase by 400%	0.3270	122,720	4.4617	23,671
	Increase by 500%	0.3270	123,142	4.4617	23,249
	Increase by 600%	0.3270	123,564	4.4617	22,827
	Increase by 700%	0.3270	123,986	4.4617	22,405
	Increase by 800%	0.3270	124,408	4.4617	21,983
	Increase by 900%	0.3270	124,830	4.4617	21,561
	Increase by 1000%	0.3270	125,252	4.4617	21,139
	Increase by 1100%	0.3270	125,674	4.4617	20,717
	Increase by 1200%	0.3270	126,097	4.4617	20,295
	Increase by 1300%	0.3270	126,519	4.4617	19,873
	Increase by 1400%	0.3270	126,941	4.4617	19,451
	Increase by 1500%	0.3270	127,363	4.4617	19,029
	Increase by 2000%	0.3270	129,473	4.4617	16,918
	Increase by 2500%	0.3270	131,583	4.4617	14,808
	Increase by 3000%	0.3270	133,694	4.4617	12,698
	Increase by 3500%	0.3270	135,804	4.4617	10,587
	Increase by 4000%	0.3270	137,914	4.4617	8,477
	Increase by 4500%	0.3270	140,025	4.4617	6,367

	**Increase by 5000%**	0.3270	142,136	4.4617	4,255
**Decrease by 20%**	**Increase by 100%**	0.3285	121,427	4.4560	24,394
	**Increase by 200%**	0.3285	121,852	4.4560	23,970
	**Increase by 300%**	0.3285	122,277	4.4560	23,545
	**Increase by 400%**	0.3285	122,701	4.4560	23,120
	**Increase by 500%**	0.3285	123,126	4.4560	22,695
	**Increase by 600%**	0.3285	123,551	4.4560	22,271
	**Increase by 700%**	0.3285	123,976	4.4560	21,846
	**Increase by 800%**	0.3285	124,400	4.4560	21,421
	**Increase by 900%**	0.3285	124,825	4.4560	20,996
	**Increase by 1000%**	0.3285	125,250	4.4560	20,572
	**Increase by 1100%**	0.3285	125,674	4.4560	20,147
	**Increase by 1200%**	0.3285	126,099	4.4560	19,722
	**Increase by 1300%**	0.3285	126,524	4.4560	19,298
	**Increase by 1400%**	0.3285	126,949	4.4560	18,873
	**Increase by 1500%**	0.3285	127,373	4.4560	18,448
	**Increase by 2000%**	0.3285	129,497	4.4560	16,325
	**Increase by 2500%**	0.3285	131,621	4.4560	14,201
	**Increase by 3000%**	0.3285	133,744	4.4560	12,077
	**Increase by 3500%**	0.3285	135,868	4.4560	9,954
	**Increase by 4000%**	0.3285	137,991	4.4560	7,830
	**Increase by 4500%**	0.3285	140,115	4.4560	5,706
	**Increase by 5000%**	0.3285	142,240	4.4560	3,582
**Decrease by 30%**	**Increase by 100%**	0.3298	121,404	4.4510	23,913
	**Increase by 200%**	0.3298	121,831	4.4510	23,486

© 2024 Chatterjee A et al. *JAMA Network Open.*

	**Increase by 300%**	0.3298	122,258	4.4510	23,059
	**Increase by 400%**	0.3298	122,685	4.4510	22,632
	**Increase by 500%**	0.3298	123,112	4.4510	22,205
	**Increase by 600%**	0.3298	123,539	4.4510	21,777
	**Increase by 700%**	0.3298	123,966	4.4510	21,350
	**Increase by 800%**	0.3298	124,393	4.4510	20,923
	**Increase by 900%**	0.3298	124,820	4.4510	20,496
	**Increase by 1000%**	0.3298	125,247	4.4510	20,069
	**Increase by 1100%**	0.3298	125,674	4.4510	19,642
	**Increase by 1200%**	0.3298	126,101	4.4510	19,215
	**Increase by 1300%**	0.3298	126,529	4.4510	18,788
	**Increase by 1400%**	0.3298	126,956	4.4510	18,361
	**Increase by 1500%**	0.3298	127,383	4.4510	17,934
	**Increase by 2000%**	0.3298	129,518	4.4510	15,798
	**Increase by 2500%**	0.3298	131,653	4.4510	13,663
	**Increase by 3000%**	0.3298	133,789	4.4510	11,528
	**Increase by 3500%**	0.3298	135,924	4.4510	9,392
	**Increase by 4000%**	0.3298	138,060	4.4510	7,257
	**Increase by 4500%**	0.3298	140,195	4.4510	5,121
	**Increase by 5000%**	0.3298	142,331	4.4510	2,985
**Decrease by 40%**	**Increase by 100%**	0.3314	121,377	4.4452	23,365
	**Increase by 200%**	0.3314	121,806	4.4452	22,936
	**Increase by 300%**	0.3314	122,236	4.4452	22,506
	**Increase by 400%**	0.3314	122,666	4.4452	22,076
	**Increase by 500%**	0.3314	123,096	4.4452	21,646

© 2024 Chatterjee A et al. *JAMA Network Open.*

	**Increase by 600%**	0.3314	123,525	4.4452	21,217
	**Increase by 700%**	0.3314	123,955	4.4452	20,787
	**Increase by 800%**	0.3314	124,385	4.4452	20,357
	**Increase by 900%**	0.3314	124,815	4.4452	19,927
	**Increase by 1000%**	0.3314	125,244	4.4452	19,498
	**Increase by 1100%**	0.3314	125,674	4.4452	19,068
	**Increase by 1200%**	0.3314	126,104	4.4452	18,638
	**Increase by 1300%**	0.3314	126,534	4.4452	18,208
	**Increase by 1400%**	0.3314	126,963	4.4452	17,779
	**Increase by 1500%**	0.3314	127,393	4.4452	17,349
	**Increase by 2000%**	0.3314	129,542	4.4452	15,200
	**Increase by 2500%**	0.3314	131,691	4.4452	13,051
	**Increase by 3000%**	0.3314	133,839	4.4452	10,903
	**Increase by 3500%**	0.3314	135,988	4.4452	8,754
	**Increase by 4000%**	0.3314	138,137	4.4452	6,605
	**Increase by 4500%**	0.3314	140,286	4.4452	4,456
	**Increase by 5000%**	0.3314	142,435	4.4452	2,307
**Decrease by 50%**	**Increase by 100%**	0.3327	121,352	4.4399	22,864
	**Increase by 200%**	0.3327	121,784	4.4399	22,432
	**Increase by 300%**	0.3327	122,216	4.4399	21,999
	**Increase by 400%**	0.3327	122,649	4.4399	21,567
	**Increase by 500%**	0.3327	123,081	4.4399	21,135
	**Increase by 600%**	0.3327	123,513	4.4399	20,703
	**Increase by 700%**	0.3327	123,945	4.4399	20,271
	**Increase by 800%**	0.3327	124,377	4.4399	19,838

© 2024 Chatterjee A et al. *JAMA Network Open.*

	**Increase by 900%**	0.3327	124,810	4.4399	19,406
	**Increase by 1000%**	0.3327	125,242	4.4399	18,974
	**Increase by 1100%**	0.3327	125,674	4.4399	18,542
	**Increase by 1200%**	0.3327	126,106	4.4399	18,110
	**Increase by 1300%**	0.3327	126,538	4.4399	17,677
	**Increase by 1400%**	0.3327	126,971	4.4399	17,245
	**Increase by 1500%**	0.3327	127,403	4.4399	16,813
	**Increase by 2000%**	0.3327	129,564	4.4399	14,652
	**Increase by 2500%**	0.3327	131,725	4.4399	12,491
	**Increase by 3000%**	0.3327	133,886	4.4399	10,330
	**Increase by 3500%**	0.3327	136,047	4.4399	8,169
	**Increase by 4000%**	0.3327	138,208	4.4399	6,008
	**Increase by 4500%**	0.3327	140,369	4.4399	3,847
	**Increase by 5000%**	0.3327	142,530	4.4399	1,685
**Decrease by 60%**	**Increase by 100%**	0.3343	121,325	4.4342	22,312
	**Increase by 200%**	0.3343	121,760	4.4342	21,877
	**Increase by 300%**	0.3343	122,195	4.4342	21,442
	**Increase by 400%**	0.3343	122,630	4.4342	21,007
	**Increase by 500%**	0.3343	123,064	4.4342	20,572
	**Increase by 600%**	0.3343	123,499	4.4342	20,138
	**Increase by 700%**	0.3343	123,934	4.4342	19,703
	**Increase by 800%**	0.3343	124,369	4.4342	19,268
	**Increase by 900%**	0.3343	124,804	4.4342	18,833
	**Increase by 1000%**	0.3343	125,239	4.4342	18,398
	**Increase by 1100%**	0.3343	125,674	4.4342	17,963

© 2024 Chatterjee A et al. *JAMA Network Open.*

	**Increase by 1200%**	0.3343	126,109	4.4342	17,528
	**Increase by 1300%**	0.3343	126,543	4.4342	17,093
	**Increase by 1400%**	0.3343	126,978	4.4342	16,659
	**Increase by 1500%**	0.3343	127,413	4.4342	16,224
	**Increase by 2000%**	0.3343	129,588	4.4342	14,049
	**Increase by 2500%**	0.3343	131,762	4.4342	11,875
	**Increase by 3000%**	0.3343	133,936	4.4342	9,701
	**Increase by 3500%**	0.3343	136,111	4.4342	7,526
	**Increase by 4000%**	0.3343	138,285	4.4342	5,352
	**Increase by 4500%**	0.3343	140,460	4.4342	3,177
	**Increase by 5000%**	0.3343	142,635	4.4342	1,002
**Decrease by 70%**	**Increase by 100%**	0.3357	121,299	4.4287	21,789
	**Increase by 200%**	0.3357	121,737	4.4287	21,352
	**Increase by 300%**	0.3357	122,174	4.4287	20,915
	**Increase by 400%**	0.3357	122,611	4.4287	20,477
	**Increase by 500%**	0.3357	123,049	4.4287	20,040
	**Increase by 600%**	0.3357	123,486	4.4287	19,602
	**Increase by 700%**	0.3357	123,924	4.4287	19,165
	**Increase by 800%**	0.3357	124,361	4.4287	18,728
	**Increase by 900%**	0.3357	124,798	4.4287	18,290
	**Increase by 1000%**	0.3357	125,236	4.4287	17,853
	**Increase by 1100%**	0.3357	125,673	4.4287	17,415
	**Increase by 1200%**	0.3357	126,111	4.4287	16,978
	**Increase by 1300%**	0.3357	126,548	4.4287	16,540
	**Increase by 1400%**	0.3357	126,986	4.4287	16,103

© 2024 Chatterjee A et al. *JAMA Network Open.*

	**Increase by 1500%**	0.3357	127,423	4.4287	15,666
	**Increase by 2000%**	0.3357	129,610	4.4287	13,478
	**Increase by 2500%**	0.3357	131,797	4.4287	11,291
	**Increase by 3000%**	0.3357	133,984	4.4287	9,104
	**Increase by 3500%**	0.3357	136,171	4.4287	6,917
	**Increase by 4000%**	0.3357	138,359	4.4287	4,730
	**Increase by 4500%**	0.3357	140,546	4.4287	2,543
	**Increase by 5000%**	0.3357	142,734	4.4287	355
**Decrease by 80%**	**Increase by 100%**	0.3372	121,272	4.4228	21,234
	**Increase by 200%**	0.3372	121,712	4.4228	20,793
	**Increase by 300%**	0.3372	122,152	4.4228	20,353
	**Increase by 400%**	0.3372	122,592	4.4228	19,913
	**Increase by 500%**	0.3372	123,032	4.4228	19,473
	**Increase by 600%**	0.3372	123,472	4.4228	19,033
	**Increase by 700%**	0.3372	123,912	4.4228	18,593
	**Increase by 800%**	0.3372	124,352	4.4228	18,153
	**Increase by 900%**	0.3372	124,793	4.4228	17,713
	**Increase by 1000%**	0.3372	125,233	4.4228	17,272
	**Increase by 1100%**	0.3372	125,673	4.4228	16,832
	**Increase by 1200%**	0.3372	126,113	4.4228	16,392
	**Increase by 1300%**	0.3372	126,553	4.4228	15,952
	**Increase by 1400%**	0.3372	126,993	4.4228	15,512
	**Increase by 1500%**	0.3372	127,433	4.4228	15,072
	**Increase by 2000%**	0.3372	129,634	4.4228	12,871
	**Increase by 2500%**	0.3372	131,835	4.4228	10,671

	**Increase by 3000%**	0.3372	134,035	4.4228	8,470
	**Increase by 3500%**	0.3372	136,236	4.4228	6,269
	**Increase by 4000%**	0.3372	138,436	4.4228	4,069
	**Increase by 4500%**	0.3372	140,637	4.4228	1,868
	**Increase by 5000%**	0.3372	142,839	4.4228	-333
**Decrease by 90%**	**Increase by 100%**	0.3387	121,245	4.4171	20,689
	**Increase by 200%**	0.3387	121,687	4.4171	20,246
	**Increase by 300%**	0.3387	122,130	4.4171	19,804
	**Increase by 400%**	0.3387	122,573	4.4171	19,361
	**Increase by 500%**	0.3387	123,016	4.4171	18,918
	**Increase by 600%**	0.3387	123,458	4.4171	18,475
	**Increase by 700%**	0.3387	123,901	4.4171	18,033
	**Increase by 800%**	0.3387	124,344	4.4171	17,590
	**Increase by 900%**	0.3387	124,787	4.4171	17,147
	**Increase by 1000%**	0.3387	125,230	4.4171	16,704
	**Increase by 1100%**	0.3387	125,672	4.4171	16,261
	**Increase by 1200%**	0.3387	126,115	4.4171	15,819
	**Increase by 1300%**	0.3387	126,558	4.4171	15,376
	**Increase by 1400%**	0.3387	127,001	4.4171	14,933
	**Increase by 1500%**	0.3387	127,443	4.4171	14,490
	**Increase by 2000%**	0.3387	129,657	4.4171	12,277
	**Increase by 2500%**	0.3387	131,871	4.4171	10,063
	**Increase by 3000%**	0.3387	134,085	4.4171	7,849
	**Increase by 3500%**	0.3387	136,299	4.4171	5,635
	**Increase by 4000%**	0.3387	138,513	4.4171	3,421

© 2024 Chatterjee A et al. *JAMA Network Open.*

	**Increase by 4500%**	0.3387	140,726	4.4171	1,207
	**Increase by 5000%**	0.3387	142,941	4.4171	-1,007

QALYs = quality-adjusted life years
ICER = incremental cost-effectiveness ratio
NMB = net monetary benefit
WTP = willingness-to-pay threshold

© 2024 Chatterjee A et al. *JAMA Network Open.*

Figure 4: Tornado Plot of Change in Net Monetary Benefit when Adjusting Key Parameters in Deterministic Sensitivity Analyses in a Closed Cohort

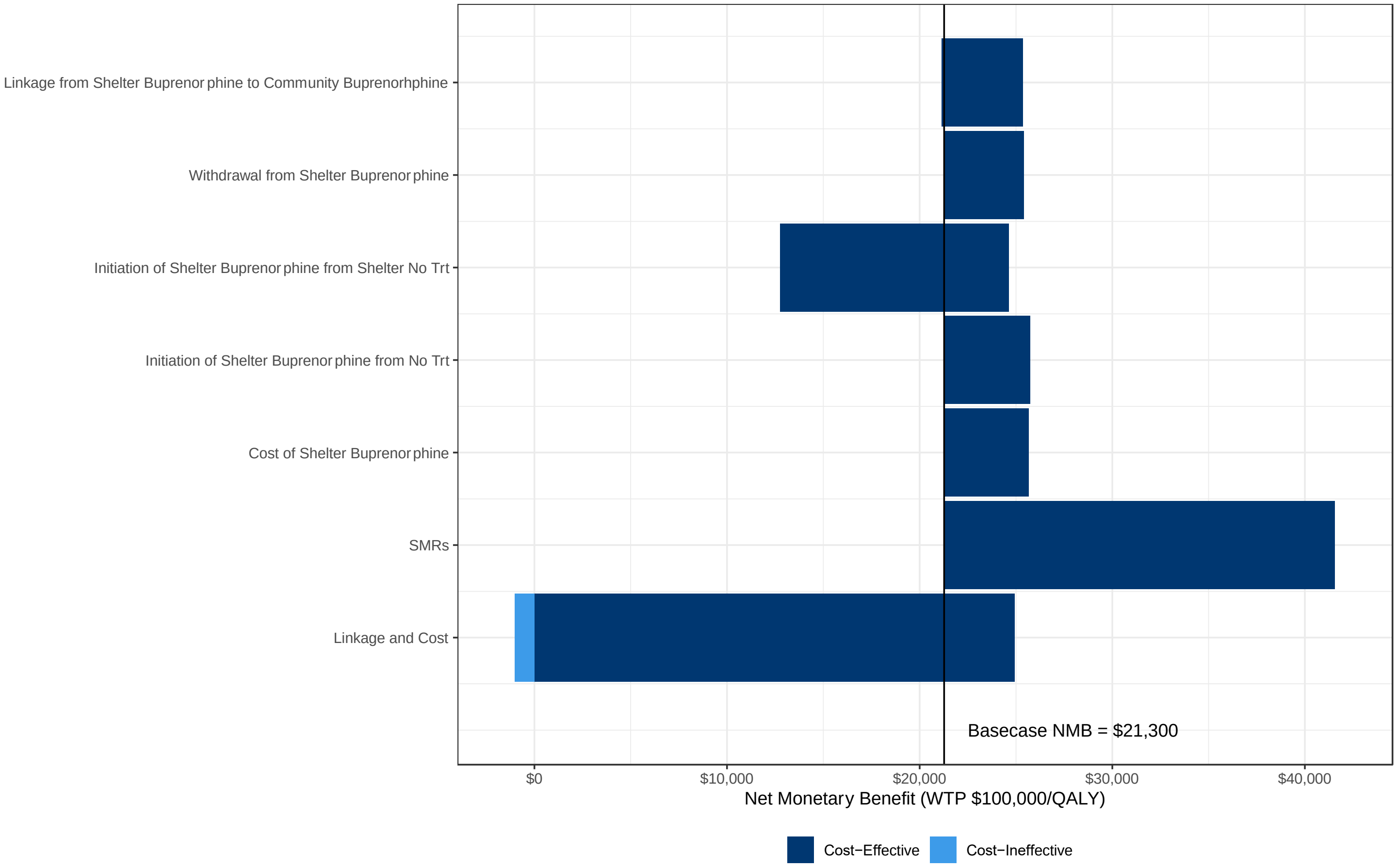

*Net Monetary Benefit is a cost-effectiveness measure that converges towards 0 for strategies that have higher cost savings but worse clinical outcomes. We used a willingness to pay (WTP) threshold of $100,000/QALY gained.

Figure legend: This figure shows net monetary benefit (NMB) results from deterministic sensitivity analyses. Parameters that were varied are included on the y-axis. The graph shows values over which the NMB was varied for each parameter evaluated, starting from a base case NMB of $21,300.

© 2024 Chatterjee A et al. *JAMA Network Open.*

Figure 5: NMB (WTP of $100,000/QALY) results from two-way deterministic sensitivity analysis on linkage to community buprenorphine from shelter buprenorphine and cost of shelter buprenorphine

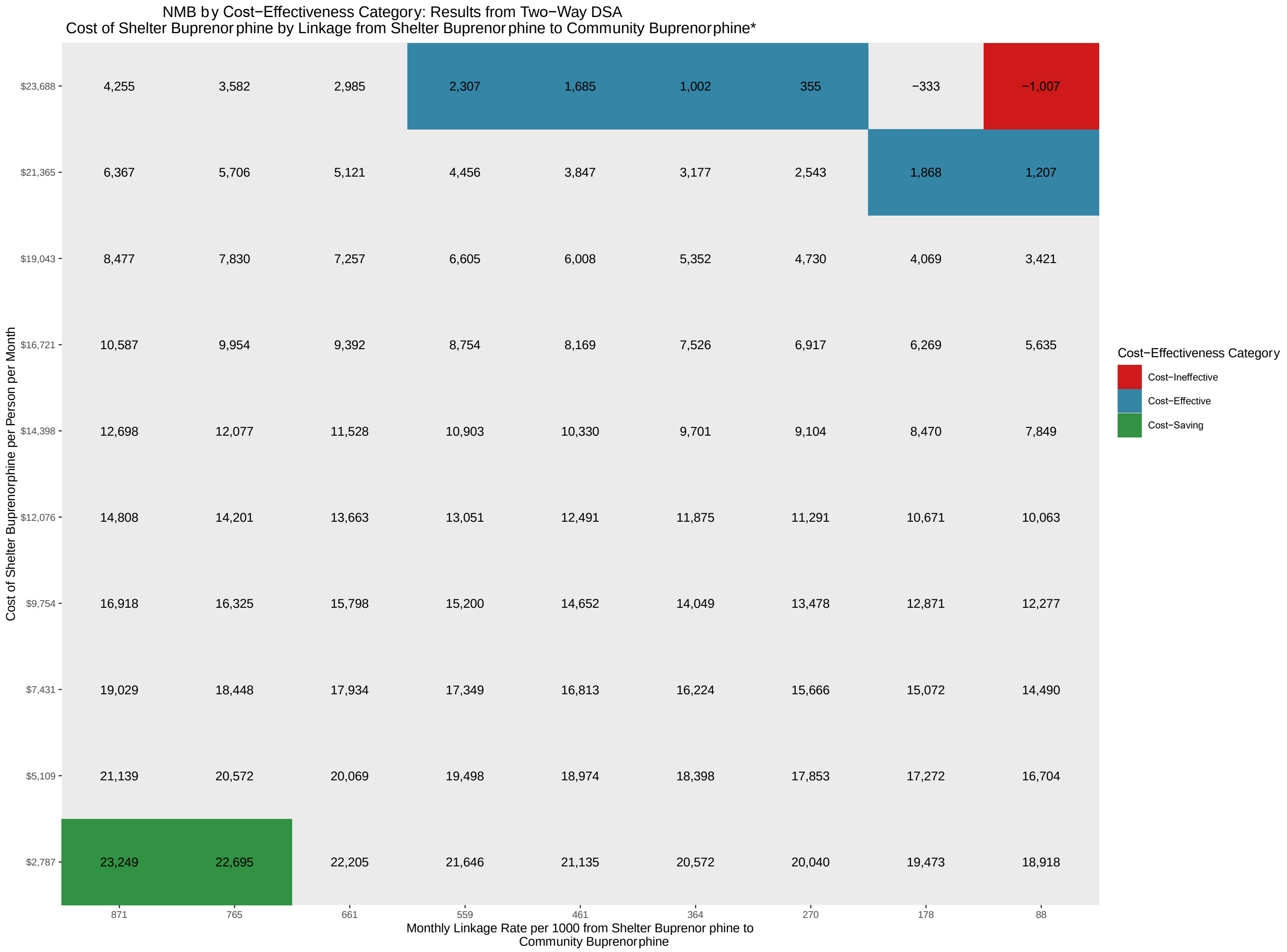

*Base case cost of shelter buprenorphine was $103 and base case monthly linkage rate per 1000 people from shelter buprenorphine to community buprenorphine was 981. Base case NMB was $21,269 at a willingness-to-pay threshold of $100,000/QALY.

© 2024 Chatterjee A et al. *JAMA Network Open.*

5.1.2 Cost-Effectiveness Acceptability Curves

Figure 6: Cost-effectiveness acceptability curve of results from a closed cohort of 13,800 PEH with OUD in MA

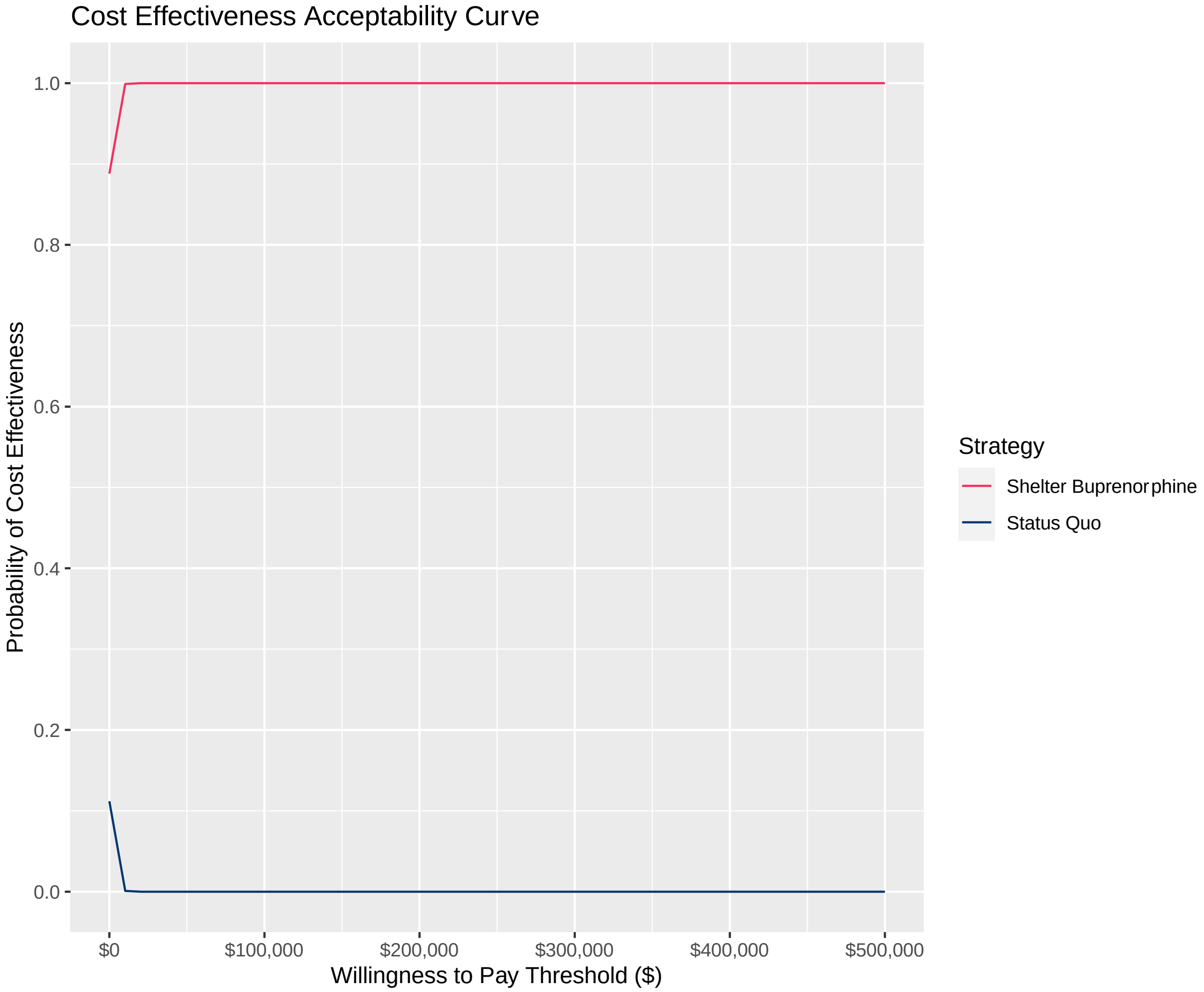

© 2024 Chatterjee A et al. *JAMA Network Open.*

Figure 7: Cost-effectiveness Acceptability Curve of results from an open cohort of PEH with OUD in MA

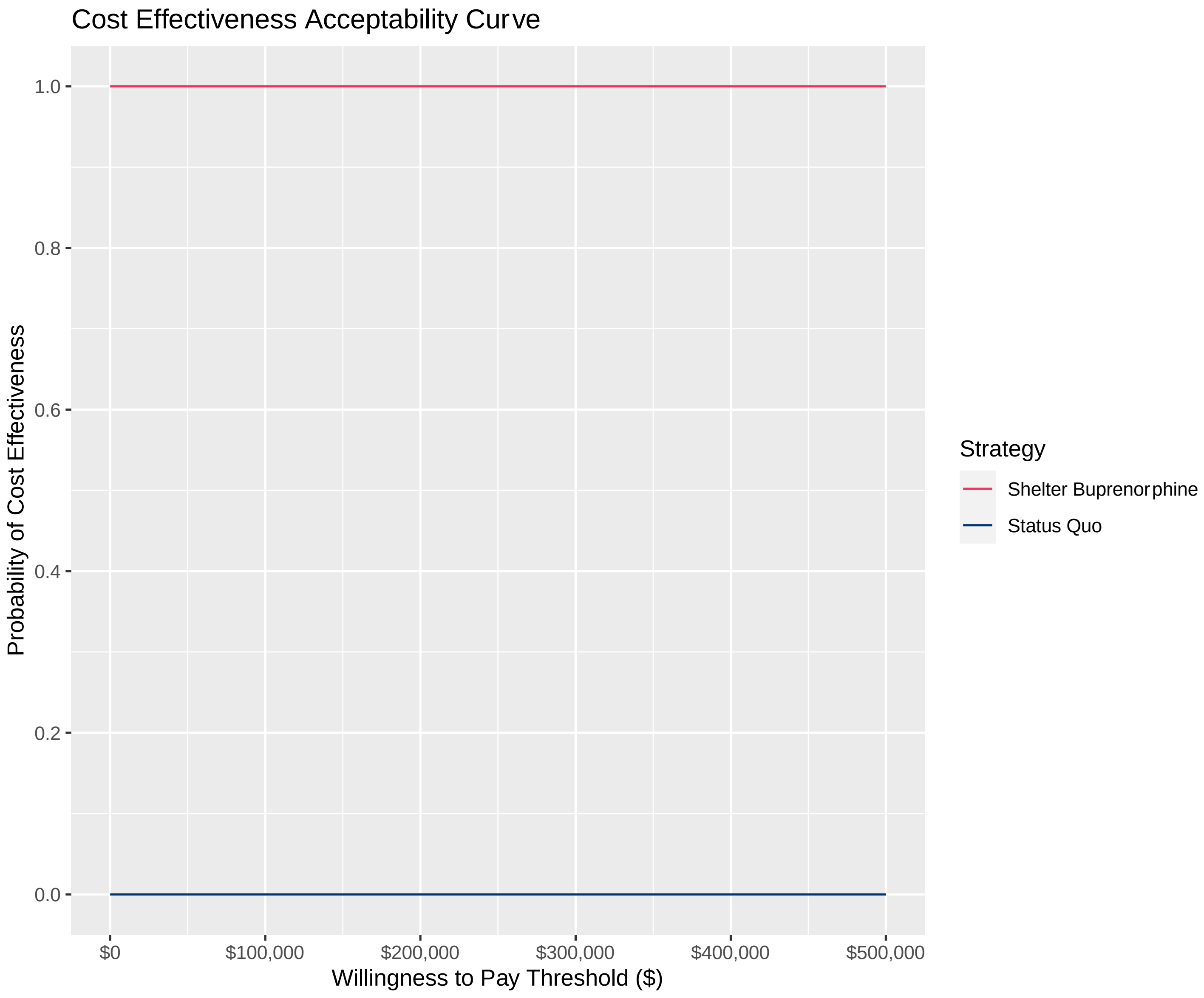

© 2024 Chatterjee A et al. *JAMA Network Open.*

eREFERENCES

1. Abuse NI on D. Drug Overdose Death Rates | National Institute on Drug Abuse (NIDA). Published June 30, 2023. Accessed March 19, 2024. https://nida.nih.gov/research-topics/trends-statistics/overdose-death-rates

2. Siebert U, Alagoz O, Bayoumi AM, et al. State-Transition Modeling: A Report of the ISPOR-SMDM Modeling Good Research Practices Task Force-3. *Value in Health*. 2012;15(6):812-820. doi:10.1016/j.jval.2012.06.014

3. Sonnenberg FA, Beck JR. Markov-Models in Medical Decision-Making - a Practical Guide. *Med Decis Making*. 1993;13(4):322-338. doi:10.1177/0272989x9301300409

4. Public Health Data Warehouse (PHD) | Mass.gov. Accessed January 11, 2023. https://www.mass.gov/public-health-data-warehouse-phd

5. Lee JD, Nunes EV Jr, Novo P, et al. Comparative effectiveness of extended-release naltrexone versus buprenorphine-naloxone for opioid relapse prevention (X:BOT): a multicentre, open-label, randomised controlled trial. *Lancet*. 2018;391(10118):309-318. doi:10.1016/S0140-6736(17)32812-X

6. Lee JD, Nunes EV, Mpa PN, et al. NIDA Clinical Trials Network CTN-0051, Extended-Release Naltrexone vs. Buprenorphine for Opioid Treatment (X:BOT): Study design and rationale. *Contemp Clin Trials*. 2016;50:253-264. doi:10.1016/j.cct.2016.08.004

7. Nunes EV, Lee JD, Sisti D, et al. Ethical and clinical safety considerations in the design of an effectiveness trial: A comparison of buprenorphine versus naltrexone treatment for opioid dependence. *Contemp Clin Trials*. 2016;51:34-43. doi:10.1016/j.cct.2016.09.006

8. Barocas JA, White LF, Wang JN, et al. Estimated Prevalence of Opioid Use Disorder in Massachusetts, 2011-2015: A Capture-Recapture Analysis. *Am J Public Health*. 2018;108(12):1675-1681. doi:10.2105/Ajph.2018.304673

9. Fine DR, Dickins KA, Adams LD, et al. Drug Overdose Mortality Among People Experiencing Homelessness, 2003 to 2018. *JAMA Netw Open*. 2022;5(1):e2142676. doi:10.1001/jamanetworkopen.2021.42676

10. HUD 2022 Continuum of Care Homeless Assistance Programs Homeless Populations and Subpopulations. Accessed March 19, 2024. https://files.hudexchange.info/reports/published/CoC_PopSub_NatlTerrDC_2022.pdf

11. Baggett TP. The CareZONE mobile outreach initiative: Pilot evaluation, 01/16/2018 - 11/16/2018. Published January 31, 2019. Accessed December 10, 2021. http://www.kraftcommunityhealth.org/wp-content/uploads/2020/04/CareZONE-evaluation-report-01-31-2019-Final-suppressed.pdf

12. Cedarbaum ER, Banta-Green CJ. Health behaviors of young adult heroin injectors in the Seattle area. *Drug Alcohol Depend*. 2016;158:102-109. doi:10.1016/j.drugalcdep.2015.11.011

13. Substance Abuse and Mental Health Services Administration R MD. 2015 National Survey on Drug Use and Health: Methodological Resource Book. *Center for Behavioral Health Statistics and Quality*. 2017;(Section 13, Statistical Inference Report).

© 2024 Chatterjee A et al. *JAMA Network Open.*

14. Neaigus A, Gyarmathy VA, Miller M, Frajzyngier VM, Friedman SR, Des Jarlais DC. Transitions to Injecting Drug Use Among Noninjecting Heroin Users: Social Network Influence and Individual Susceptibility. *JAIDS Journal of Acquired Immune Deficiency Syndromes*. 2006;41(4):493-503. doi:10.1097/01.qai.0000186391.49205.3b

15. Shah NG, Galai N, Celentano DD, Vlahov D, Strathdee SA. Longitudinal predictors of injection cessation and subsequent relapse among a cohort of injection drug users in Baltimore, MD, 1988-2000. *Drug Alcohol Depend*. 2006;83(2):147-156. doi:10.1016/j.drugalcdep.2005.11.007

16. Nosyk B, Li L, Evans E, et al. Characterizing longitudinal health state transitions among heroin, cocaine, and methamphetamine users. *Drug Alcohol Depend*. 2014;140:69-77. doi:10.1016/j.drugalcdep.2014.03.029

17. Bailey GL, Herman DS, Stein MD. Perceived relapse risk and desire for medication assisted treatment among persons seeking inpatient opiate detoxification. *J Subst Abuse Treat*. 2013;45(3):302-305. doi:10.1016/j.jsat.2013.04.002

18. Hunink M, Weinstein M, Wittenberg E, et al. *Decision Making in Health and Medicine: Integrating Evidence and Values, Second Edition*.; 2014.

19. Neumann PJ, Ganiats TG, Russell LB, Sanders GD, Siegel JE, eds. *Cost-Effectiveness in Health and Medicine*. Oxford University Press; 2016. doi:10.1093/acprof:oso/9780190492939.001.0001

20. Jiang R, Janssen MFB, Pickard AS. US population norms for the EQ-5D-5L and comparison of norms from face-to-face and online samples. *Qual Life Res*. 2021;30(3):803-816. doi:10.1007/s11136-020-02650-y

21. Wittenberg E, Bray JW, Aden B, Gebremariam A, Nosyk B, Schackman BR. Measuring benefits of opioid misuse treatment for economic evaluation: health-related quality of life of opioid-dependent individuals and their spouses as assessed by a sample of the US population. *Addiction*. 2016;111(4):675-684. doi:10.1111/add.13219

22. Murphy SM, McCollister KE, Leff JA, et al. Cost-Effectiveness of Buprenorphine–Naloxone Versus Extended-Release Naltrexone to Prevent Opioid Relapse. *Ann Intern Med*. 2019;170(2):90-98. doi:10.7326/M18-0227

23. McCollister KE, Leff JA, Yang X, et al. Cost of Pharmacotherapy for Opioid Use Disorders Following Inpatient Detoxification. *Am J Manag Care*. 2018;24(11):526-531.

24. Social Workers : Occupational Outlook Handbook: : U.S. Bureau of Labor Statistics. Accessed November 21, 2023. https://www.bls.gov/ooh/community-and-social-service/social-workers.htm#tab-5

25. Social and Community Service Managers : Occupational Outlook Handbook: : U.S. Bureau of Labor Statistics. Accessed November 21, 2023. https://www.bls.gov/ooh/management/social-and-community-service-managers.htm#tab-5

26. Han BH, Doran KM, Krawczyk N. National trends in substance use treatment admissions for opioid use disorder among adults experiencing homelessness. *Journal of Substance Abuse Treatment*. 2022;132. doi:10.1016/j.jsat.2021.108504

© 2024 Chatterjee A et al. *JAMA Network Open.*

27. Chatterjee A, Weitz M, Savinkina A, et al. Estimated Costs and Outcomes Associated With Use and Nonuse of Medications for Opioid Use Disorder During Incarceration and at Release in Massachusetts. *JAMA Network Open*. 2023;6(4):e237036. doi:10.1001/jamanetworkopen.2023.7036

28. Cannata NC. Massachusetts Department of Correction. Published online 2019:64.

29. Bertram W, Jones A. How many people in your state go to local jails every year? :4.

30. Jackson C. Multi-State Models for Panel Data: The msm Package for R. *2011*. 2011;38(8):28. doi:10.18637/jss.v038.i08

31. Morgan JR, Schackman BR, Leff JA, Linas BP, Walley AY. Injectable naltrexone, oral naltrexone, and buprenorphine utilization and discontinuation among individuals treated for opioid use disorder in a United States commercially insured population. *J Subst Abuse Treat*. 2018;85:90-96. doi:10.1016/j.jsat.2017.07.001

32. Strain EC, Stitzer ML, Liebson IA, Bigelow GE. Dose-response effects of methadone in the treatment of opioid dependence. *Annals of Internal Medicine*. 1993;119(1):23-27.

33. Morgan JR, Schackman BR, Weinstein ZM, Walley AY, Linas BP. Overdose following initiation of naltrexone and buprenorphine medication treatment for opioid use disorder in a United States commercially insured cohort. *Drug and Alcohol Dependence*. 2019;200:34-39. doi:10.1016/j.drugalcdep.2019.02.031

34. Sordo L, Barrio G, Bravo MJ, et al. Mortality risk during and after opioid substitution treatment: systematic review and meta-analysis of cohort studies. *BMJ*. 2017;357:j1550. doi:10.1136/bmj.j1550

35. O'Driscoll PT, McGough J, Hagan H, Thiede H, Critchlow C, Alexander ER. Predictors of accidental fatal drug overdose among a cohort of injection drug users. *Am J Public Health*. 2001;91(6):984-987.

36. Zeger SL, Liang KY. Longitudinal Data Analysis for Discrete and Continuous Outcomes. *Biometrics*. 1986;42(1):121-130. doi:10.2307/2531248

37. National Center for Health Statistics. National Vital Statistics System: Mortality Multiple Cause-of-Death. Published online 2013. https://www.cdc.gov/nchs/nvss/mortality_public_use_data.htm

38. Centers for Disease Control and Prevention. *HIV Infection Risk, Prevention, and Testing Behaviors among Persons Who Inject Drugs—National HIV Behavioral Surveillance: Injection Drug Use, 23 U.S. Cities, 2018.*; 2020. http://www.cdc.gov/hiv/library/reports/hivsurveillance.html

39. Cedarbaum ER, Banta-Green CJ. Health behaviors of young adult heroin injectors in the Seattle area. *Drug and Alcohol Dependence*. 2016;158:102-109. doi:10.1016/j.drugalcdep.2015.11.011

40. Roncarati JS, Baggett TP, O'Connell JJ, et al. Mortality Among Unsheltered Homeless Adults in Boston, Massachusetts, 2000-2009. *JAMA Internal Medicine*. 2018;178(9):1242. doi:10.1001/jamainternmed.2018.2924

41. Morgan JR, Schackman BR, Leff JA, Linas BP, Walley AY. Injectable naltrexone, oral naltrexone, and buprenorphine utilization and discontinuation among individuals treated for opioid use disorder in a United States commercially insured population. *J Subst Abuse Treat*. 2018;85:90-96. doi:10.1016/j.jsat.2017.07.001

42. Bailey GL, Herman DS, Stein MD. Perceived relapse risk and desire for medication assisted treatment among persons seeking inpatient opiate detoxification. *J Subst Abuse Treat*. 2013;45(3):302-305. doi:10.1016/j.jsat.2013.04.002

© 2024 Chatterjee A et al. *JAMA Network Open.*
