# Supplementary material for: Health and Economic Outcomes of Offering Buprenorphine in Homeless Shelters in Massachusetts
Source: JAMA Netw Open. 2024 Oct 16;7(10):e2437233. doi: 10.1001/jamanetworkopen.2024.37233 (PMC11581564; doi:10.1001/jamanetworkopen.2024.37233)
Supplement: Supplement 2. — Data Sharing Statement [file jamanetwopen-e2437233-s002.pdf]

Data Sharing Statement

Chatterjee. Health and Economic Outcomes of Offering Buprenorphine in Homeless Shelters in Massachusetts. *JAMA Netw Open*. Published October 03, 2024.
doi:10.1001/jamanetworkopen.2024.37233

Data
Data available: No

Additional Information
Explanation for why data not available: Model documentation is available on the syndemics lab (https://www.syndemicslab.org/) website. We are working through licensing concerns to make code for the model to be available on a public site like git-hub.
